# Scaling Exponents of Time Series Data: A Machine Learning Approach

**DOI:** 10.3390/e25121671

**Published:** 2023-12-18

**Authors:** Sebastian Raubitzek, Luiza Corpaci, Rebecca Hofer, Kevin Mallinger

**Affiliations:** 1 Information and Software Engineering Group, TU Wien, Favoritenstrasse 9-11/194, 1040 Vienna, Austria; 2 SBA Research gGmbh, Floragasse 7, 1040 Vienna, Austria; 3 Research Group Security and Privacy, University of Vienna, Kolingasse 14-16, 1090 Vienna, Austria

**Keywords:** scaling exponent, Hurst exponent, machine learning, artificial intelligence, complexity, regression analysis

## Abstract

In this study, we present a novel approach to estimating the Hurst exponent of time series data using a variety of machine learning algorithms. The Hurst exponent is a crucial parameter in characterizing long-range dependence in time series, and traditional methods such as Rescaled Range (R/S) analysis and Detrended Fluctuation Analysis (DFA) have been widely used for its estimation. However, these methods have certain limitations, which we sought to address by modifying the R/S approach to distinguish between fractional Lévy and fractional Brownian motion, and by demonstrating the inadequacy of DFA and similar methods for data that resembles fractional Lévy motion. This inspired us to utilize machine learning techniques to improve the estimation process. In an unprecedented step, we train various machine learning models, including LightGBM, MLP, and AdaBoost, on synthetic data generated from random walks, namely fractional Brownian motion and fractional Lévy motion, where the ground truth Hurst exponent is known. This means that we can initialize and create these stochastic processes with a scaling Hurst/scaling exponent, which is then used as the ground truth for training. Furthermore, we perform the continuous estimation of the scaling exponent directly from the time series, without resorting to the calculation of the power spectrum or other sophisticated preprocessing steps, as done in past approaches. Our experiments reveal that the machine learning-based estimators outperform traditional R/S analysis and DFA methods in estimating the Hurst exponent, particularly for data akin to fractional Lévy motion. Validating our approach on real-world financial data, we observe a divergence between the estimated Hurst/scaling exponents and results reported in the literature. Nevertheless, the confirmation provided by known ground truths reinforces the superiority of our approach in terms of accuracy. This work highlights the potential of machine learning algorithms for accurately estimating the Hurst exponent, paving new paths for time series analysis. By marrying traditional finance methods with the capabilities of machine learning, our study provides a novel contribution towards the future of time series data analysis.

## 1. Introduction

The Hurst (or scaling) exponent, a key parameter in time series analysis, quantifies the long-range dependence and persistence in the underlying processes [1]. Accurate estimation of the Hurst exponent is crucial for understanding and modelling various phenomena in diverse fields, such as finance [2], geophysics [3], and biomedicine [4]. Traditional methods, such as R/S analysis [5] and Detrended Fluctuation Analysis (DFA) [6], have been extensively employed for this purpose. However, these techniques have certain limitations, such as sensitivity to non-stationarity and estimation biases [7,8], which has motivated researchers to explore alternative approaches to improve estimation accuracy and robustness [9]. Further, these techniques do not apply well to short time series data or when studying small sliding window sizes to obtain a dynamic estimate of the scaling exponent. As a rule of thumb, when we talk about short time series data, we mean a dataset with around 100 or fewer samples.

Machine learning algorithms have shown remarkable success in various domains, including time series prediction [10] and feature extraction [11], presenting an intriguing opportunity to address the challenges associated with estimating the Hurst exponent. Therefore, the research question we aim to address in this paper is: can machine learning models outperform traditional methods in estimating the Hurst exponent of time series data?

In this paper, we investigate the application of several machine learning models, including LightGBM [12], MLP, and AdaBoost [13], to predict the Hurst exponent of time series data. We train these models on synthetic data generated from random walks, i.e., fractional Brownian motion [14] and *fractional Lévy motion* [15], where the ground truth Hurst/scaling exponent is known. This allows us to rigorously evaluate the performance of the machine learning-based estimators in comparison to traditional methods/algorithms such as R/S analysis [5] and Detrended Fluctuation Analysis (DFA) [6].

Our experimental results demonstrate that the proposed machine learning models outperform the traditional techniques in estimating the Hurst exponent. Furthermore, we apply our trained models to real-world financial data and observe that the estimated Hurst exponents are contradictory with the values reported in the literature. However we also provide evidence, that the data under study rather follows a fractional Lévy than a fractional Brownian motion, which in simple terms means that it allows for extreme events and, further, that the fluctuations are not uniform across different scales [16]. We further show that the trained machine learning models are particularly well suited to estimating the scaling exponents of these stochastic processes. These findings suggest that machine learning algorithms have the potential to serve as effective tools for estimating the Hurst exponent, providing new insights into the analysis of time series data, and are capable of outperforming traditional methods.

The present study contributes to the existing body of knowledge in the following ways:

- We present a novel modification to the R/S approach, highlighting the distinctions between fractional Lévy motions, fractional Brownian motions, and stock market data.
- We introduce a method for continuously estimating a scaling parameter via machine learning from time series data without employing sophisticated preprocessing methods.
- We propose a new technique for estimating the scaling exponent of fractional Lévy motion using machine learning models, demonstrating its effectiveness through extensive experiments.
- We show that traditional techniques like DFA and other traditional methods do not accurately depict the scaling parameter for time series that are close to fractional Lévy motion, emphasizing the potential for machine learning approaches in this realm.

The organization of this article is as follows:

Section 2 offers an overview of approaches similar to ours and discusses related works to the present article.

Section 3 gives a quick introduction to the Hurst exponent, traditional methods of estimation, and the machine learning algorithms used in this study.

Section 4 presents a detailed description of the training and validation process of the machine learning models used in this work. Furthermore, we outline the process of synthetic data generation and discuss the pros and cons of the trained machine learning models for estimating the scaling parameter of a time series. We then apply these trained machine learning models in Section 5 to estimate the scaling exponent of three different financial time series data, comparing our results with those found in the literature.

In Section 5.3, we provide a summary and discussion of our experiments and ideas. Our findings are then concluded in Section 6. To maintain the focus of the main text and to provide additional results that further emphasize our findings, we have included Appendix A and Appendix B.

## 2. Related Work

There is a limited number of publications on the estimation of the Hurst exponent through machine learning. Ledesmann et al. [9] initially proposed estimating the Hurst exponent via Artificial Neural Networks (ANN), employing a feature extraction method based on the power spectra of the data series used for network training. To do this, the authors generated a training dataset consisting of five classes with 1000 training instances each, where each class corresponds to a Hurst exponent value within the range of 0.5 < *H* < 1.0. They computed the relative power values $P_{i}$ from the training datasets to construct the network’s training set. A comparative analysis with other standard prediction methods reveals that using ANN produces accurate predictions of the Hurst exponent. However, the authors do not specify whether the datasets were generated using Brownian or Lévy motion, nor do they explain their decision to exclude Hurst exponents below 0.5 in their study.

Subsequent studies by Mukherjee et al. [17] and Sadhukhan et al. [18] also adopt this method, where the later applying it to earthquake dataset analysis. They utilize moving average smoothing to extract features from input time series data. The estimated Hurst exponent results are compared with other signal processing methods, exhibiting similar outcomes. Mukherjee et al. [17] apply the method to two datasets, one comprising national stock exchange data and the other consisting of network traffic statistics. They employ an exponentially weighted moving average filter to extract features from input time series, yielding ten features, one from each filter. These feature vectors are then used for network training. The Hurst exponent of each time series in the dataset is predicted by feeding it into the network. The Hurst exponent values are first estimated through standard statistical signal processing methods, followed by the neural network’s determination of the mapping between the time series and corresponding Hurst exponent. Their datasets contain Hurst exponents within the range of 0 < *H* < 1. The ANN-derived Hurst exponent values closely resemble those obtained through DFA and RS methods, with the authors noting that ANN proves faster than alternative techniques.

Tarnopolski [19] explores a distinct approach to estimating the Hurst exponent through machine learning. They identify a correlation between the maximal Lyapunov exponent and the Hurst exponent, subsequently employing the former quantity to predict the latter via Nearest Neighbour. This requires several thousand values of both exponents for training, followed by the use of $2\times 10^{5}$ Lyapunov exponents for prediction. This finding is intriguing, as the interpretation of these two values would intuitively point in opposite directions: Lyapunov exponent measures sensitivity to initial conditions, while the Hurst exponent quantifies persistency.

Tyralis et al. [20] apply random forest and linear regression algorithms to compute the Hurst exponent based on real-world precipitation data, and also found that the machine learning methods yield similar results as the standard methods. Meanwhile, Bulkah et al. [21] compare classification against regression trees for predicting the Hurst exponent, working with artificially generated time series. The training time series are obtained through the generation of multifractal cascades with weights, containing Hurst exponents within the range of 0.5 < *H* < 1 and partitioned into classes according to *H* values. Each training sample ranges in length from 512 to 4096 data points. The results indicate that the correct class determination probability increases with input length. The highest accuracy is achieved for a training set of 4096 data points using regression trees.

## 3. Methodology

This paper synergistically integrates a variety of methodologies and concepts to yield innovative insights. Firstly, it delves into stochastic processes, such as fractional Brownian motion, a prominent example of random walks. Secondly, it explores estimation techniques, specifically focusing on the determination of the probability characteristics, such as the Hurst exponent, for random walks and, by extension, arbitrary time series data. Lastly, the study harnesses the power of Machine Learning to further enhance the analytical capabilities of the aforementioned methods.

### 3.1. Random Walks

Stochastic processes are a fundamental concept in probability theory and statistical analysis, representing the evolution of random variables over time or space. As opposed to deterministic processes, which follow a fixed set of rules or equations, stochastic processes are characterized by their probabilistic nature, allowing for a rich and flexible modelling framework. These processes encompass a wide range of phenomena, from natural systems to financial markets, where inherent randomness and uncertainty give rise to complex behaviors and patterns.

Here, we focus on the parametrization of two specific stochastic processes, the fractional Brownian motion (fBm), and fractional Lévy motion (fLm), which extend the well-known Brownian motion and Lévy motion by introducing self-similarity and long-range dependence, capturing more complex behaviors observed in various fields. For a detailed discussion of these stochastic processes, the interested reader is referred to the work of Liu et al. (2020) and Huillet (1999) [22,23].

Starting with the fBm, it is important to note that it is an extension of standard Brownian motion where increments, while still Gaussian, are no longer independent. Instead, they can be correlated, with the degree of correlation captured by the Hurst parameter, *H*. The autocorrelation function of fBm can be given by:

(1) $$\gamma \left(k\right)=\frac{\sigma^{2}}{2}\left[{\left|k-1\right|}^{2H}-{2|k|}^{2H}+{\left|k+1\right|}^{2H}\right]\;.$$

Here, σ is the width of the Gaussian distribution of increments, and *H* is the Hurst parameter. When H=0.5, we recover standard Brownian motion with independent increments. Increments are negatively correlated when H<0.5, and positively correlated when H>0.5.

On the other hand, we have the fLm, which extends upon fBm by replacing Gaussian-distributed increments with Lévy stable distributions, thus accommodating a wider range of increment distributions to better model certain complex systems. A Lévy stable distribution is characterized by its Fourier transform:

(2) $$p_{\alpha ,\beta }\left(k;\mu ,\sigma \right)=\exp\left[i\mu k-\sigma^{\alpha }{\left|k\right|}^{\alpha }\left(1-i\beta k\mathrm{sign}(k)\omega (k,\alpha )\right)\right]\;,$$

where

(3) $$\omega \left(k,\alpha \right)=\left\{\begin{array}{cc} \tan\left(\frac{\pi \alpha }{2}\right) & \mathrm{if}\;\alpha \neq 1,0<\alpha <2,\\ -\frac{2}{\pi }\ln\left|k\right| & \mathrm{if}\;\alpha =1. \end{array}\right.\;.$$

Here, α is the Lévy index determining the thickness of the tails of the distribution (0 < α≤2), β is the skewness parameter, μ is the shift parameter, and σ is a scale parameter.

In fLm, the Hurst parameter *H* is introduced via a deterministic kernel function in the following stochastic integral, capturing the dependence structure of increments:

(4) $$L_{H}\left(t\right)=\int_{0}^{t}K_{H}\left(t,s\right)dL\left(s\right)\;.$$

In this equation (stochastic integral), $K_{H}\left(t\right)$ is the deterministic kernel function depending on the Hurst exponent *H* and the characteristic exponent Ψ(u) of the underlying Lévy process. When H=0.5, the process shows independent increments. When H<0.5, increments of opposite signs are more likely to cluster together. When H>0.5, increments of the same sign are more likely to cluster together.

Overall, both *H* and α play significant roles in the characterization of fBm and fLm:

The Hurst parameter *H*, common to both fBm and fLm, captures the correlation or the memory effect between increments in these processes. The behavior of the process changes from independent increments at H=0.5, to anti-persistent at H<0.5, and persistent at H>0.5.

The Lévy index α is specific to fLm and governs the thickness of the tails of the underlying increment distribution. For α=2, the increment distribution is Gaussian, reducing the process to fBm. When 0<α≤2, the distribution has infinite variance and a heavy tail, with lower α leading to heavier tails and more frequent extreme events. For α=2 The Lévy distribution reduces to the Gaussian case.

However, in the definition of the fLm via the stochastic integral (Equation (4)), the Lévy increments directly enter the picture without explicit reference to their characteristic function. This is somewhat similar to how one defines a Brownian motion by integrating Gaussian increments, without directly referring to the Gaussian distribution in the integral [24].

To summarize, the distribution of Lévy increments (including their characteristic function) and the kernel function enter the picture in different places when dealing with a fLm. The kernel function is part of the definition of the fLm, modulating how increments of the underlying Lévy motion contribute to the fLm. The distribution of Lévy increments is, of course, crucial for determining the properties of the underlying Lévy motion, and it also comes into play when one computes quantities related to the fLm (like its autocorrelation function).

With the capability to modulate *H* and α, both fBm and fLm offer a comprehensive framework to model a variety of complex behaviors observed in real-world phenomena, including finance (e.g., modeling stock prices), physics, geophysics, and network traffic analysis. As such, understanding and tuning these parameters is crucial to successfully harnessing these stochastic processes for practical applications.

For our simulations we used the Pyhton package hurst to simulate fractional Brownian motions and the code provide by [25], which is based on the algorithm from [26]. for our purposes we varied only the Hurst/scaling parameter for the fractional Brownian motions and the Hurst/scaling parameter and the Lévy index α for the fractional Lévy motions. Apart from that we went with the default values of the employed code.

### 3.2. Estimating the Hurst Exponent

The Hurst exponent, denoted as *H*, is a key parameter in time series analysis, as it quantifies the long-range dependence and persistence in the underlying processes [1]. Fractional Brownian motion (fBm), introduced by Mandelbrot and Van Ness [14], is a widely used model for processes exhibiting long-range dependence, and the Hurst exponent is a crucial parameter in characterizing fBm. Estimating the Hurst exponent from time series data can provide valuable insights into the nature of the data and the underlying process.

To estimate the Hurst exponent from time series data, several methods have been proposed, with Rescaled Range (R/S) analysis [5] and Detrended Fluctuation Analysis (DFA) [6] being two of the most popular techniques. Both methods are based on the idea of analyzing the scaling behavior of the data at different time scales, and the Hurst exponent is then inferred from the relationship between the analyzed quantities and the time scales.

#### 3.2.1. R/S Analysis

The R/S analysis, introduced by Harold E. Hurst, calculates the range of cumulative deviations of the time series data, rescaled by the standard deviation of the data. This process is repeated for various time scales, and the Hurst exponent is estimated from the slope of the log–log plot of the rescaled range versus the time scale.

Rescaled Range (R/S) analysis is a widely used method for estimating the Hurst exponent (*H*) from time series data, which characterizes the long-range dependence and persistence in the underlying processes. R/S analysis was introduced by Harold E. Hurst in his seminal work on the long-term storage capacity of reservoirs [5].

To perform R/S analysis, the following steps are taken:

Given a time series $X_{t}$ of length *N*, compute the mean $\bar{X}$. Calculate the mean-adjusted time series $Y_{t}$ by subtracting the mean from each data point: $Y_{t}=X_{t}-\bar{X}$. Compute the cumulative deviation series $Z_{t}$ by summing the mean-adjusted time series up to time *t*: $Z_{t}=\sum_{1}^{t}\left(Y_{i}\right)$. For each non-overlapping subseries of length *n* (n<N), compute the range R(n) as the difference between the maximum and minimum values of the cumulative deviation series within that subseries. Calculate the standard deviation S(n) for each subseries of length *n*. Compute the rescaled range $\frac{R(n)}{S(n)}$ by dividing the range R(n) by the standard deviation S(n) for each subseries of length *n*. For various values of *n*, calculate the average $\frac{R(n)}{S(n)}$ across all subseries of length *n*. Plot the log–log relationship between the average $\frac{R(n)}{S(n)}$ and the subseries of length *n*. Estimate the Hurst exponent (H) as the slope of the linear regression line fitted to the log–log plot. The R/S analysis is based on the assumption that the rescaled range $\frac{R(n)}{S(n)}$ scales with the subseries of length n through a power–law relationship: $\frac{R(n)}{S(n)}\approx n^{H}$. Therefore, the Hurst exponent can be estimated from the slope of the log–log plot of $\frac{R(n)}{S(n)}$ versus n. The estimated Hurst exponent provides insights into the long-range dependence and persistence of the time series data, with 0<H<1.

For our analysis we used the Python packages nolds and hurst, whereas the hurst package also provides a simplified version of the R/S analysis.

#### 3.2.2. Detrended Fluctuation Analysis (DFA)

Detrended Fluctuation Analysis (DFA) is a widely used technique for detecting long-range correlations in non-stationary time series data. The method was initially proposed by Peng et al. in their 1994 paper titled “Mosaic Organization of DNA Nucleotides” [6]. The main idea of DFA is to investigate the scaling behavior of a time series by analyzing the fluctuation function after detrending the data.

Here is an outline of the DFA procedure:

1. Integrate the time series: Calculate the cumulative sum of the deviations of the data points from their mean.
2. Divide the integrated time series into non-overlapping segments of equal length n.
3. Detrend the data: In each segment, fit a polynomial function (usually a linear function) and subtract it from the integrated time series.
4. Calculate the root-mean-square fluctuations for each segment.
5. Average the fluctuations over all segments and obtain the fluctuation function F(n).
6. Repeat steps 2–5 for various time scales (segment lengths) *n*.
7. Analyze the scaling behavior of F(n) by plotting it against the time scale *n* on a log–log scale. A linear relationship indicates the presence of long-range correlations in the original time series.
8. The Hurst exponent can be estimated from the slope of the log–log plot, providing information about the persistence or anti-persistence in the time series.

In this article, we employed the detrended fluctuation analysis from the python package nolds.

### 3.3. Machine Learning

In this section, we provide an overview of the machine learning algorithms utilized in our experiments, encompassing a diverse array of approaches to ensure a comprehensive assessment. The algorithms we implement can be categorized into three primary classes: linear models, boost regressors, and multi layer perceptrons.

By leveraging these distinct machine learning approaches, we aim to provide a thorough investigation of their efficacy in estimating the Hurst exponent for time series data, ultimately informing the development of a reliable and robust estimation method.

#### 3.3.1. Linear Models

In our study, we used two linear models, i.e., extensions to the classical linear regression, i.e., Lasso and Ridge regression.

Lasso (least absolute shrinkage and selection operator) and ridge regression are two regularization techniques used to improve the performance of linear regression models and prevent overfitting. They introduce penalty terms to the linear regression objective function, effectively constraining the magnitude of the model’s coefficients. The main difference between Lasso and Ridge regression lies in the penalty terms they use.

Ridge regression, in Ref. [27], adds an L2-norm penalty term to the linear regression objective function, which is the sum of the squared coefficients. This encourages the model to have smaller coefficients, reducing the complexity of the model and making it less prone to overfitting. The objective function of Ridge regression is:

(5) $$L\left(w\right)=\sum {\left(y_{i}-w^{T}*x_{i}\right)}^{2}+{\lambda *||w||}^{2}\;.$$

Here, L(w) is the objective function, $y_{i}$ represents the actual target value, $x_{i}$ is the feature vector, *w* is the coefficient vector, and λ is the regularization parameter controlling the strength of the penalty term. The term ${\left||w|\right|}^{2}$ denotes the squared L2-norm of the coefficient vector *w*.

Lasso regression, in Ref. [28], adds an L1-norm penalty term to the linear regression objective function, which is the sum of the absolute values of the coefficients. This not only encourages smaller coefficients but also promotes sparsity in the model, effectively performing feature selection by driving some coefficients to zero. The objective function of Lasso regression is:

(6) $$L\left(w\right)=\sum {\left(y_{i}-w^{T}*x_{i}\right)}^{2}+{\lambda *||w||}_{1}\;.$$

Here, L(w) is the objective function, $y_{i}$ represents the actual target value, $x_{i}$ is the feature vector, *w* is the coefficient vector, and λ is the regularization parameter controlling the strength of the penalty term. The term ${\left||w|\right|}_{1}$ denotes the L1-norm of the coefficient vector *w*, which is the sum of the absolute values of the coefficients.

#### 3.3.2. Boost Regressors

Boosting in machine learning is an ensemble technique used to improve the performance of weak learners (models) by combining them into a single, more accurate, and robust model. The main idea behind boosting is to iteratively train a series of weak learners on the data, with each learner focusing on correcting the errors made by its predecessor. This process encourages the models to learn from each other and compensate for their individual weaknesses, ultimately leading to a stronger, more accurate ensemble model.

- **AdaBoost:**
  AdaBoost, short for “Adaptive Boosting”, is a popular ensemble learning algorithm used in machine learning. It was developed to improve the performance of weak classifiers by combining them into a single, more accurate and robust classifier. The main idea behind AdaBoost is to iteratively train a series of weak classifiers on the data, assigning higher weights to misclassified instances at each iteration. This process encourages the subsequent classifiers to focus on the more challenging instances, ultimately leading to an ensemble model with an improved overall performance [13].
- **CatBoost:**
  CatBoost is a gradient boosting algorithm specifically designed to handle categorical features effectively. It was developed by Yandex researchers and engineers, and it is known for its high performance and accuracy in various machine learning tasks. CatBoost addresses the common challenges associated with handling categorical features, such as one-hot encoding, by employing an efficient, target-based encoding scheme called “ordered boosting”. This method reduces overfitting and improves generalization, leading to better results in many applications [29].
- **LightGBM:**
  LightGBM (Light Gradient Boosting Machine) is a gradient boosting framework developed by Microsoft that is designed to be more efficient and scalable than traditional gradient boosting methods. It is particularly well-suited for large-scale and high-dimensional data. LightGBM incorporates several key innovations, such as Gradient-based One-Side Sampling (GOSS) and Exclusive Feature Bundling (EFB), which significantly reduce memory usage and computational time while maintaining high accuracy [12].

#### 3.3.3. Multi Layer Perceptron

This research uses a Multi Layer Perceptron as a fully connected feedforward artificial neural network for regression from scikit-learn [30]. It consists of an input layer, hidden layer, and output layer that vary in size based on the hyperparameter settings. The perceptrons in the hidden and output layer inhibit a nonlinear activation function that are used to update the weights of the neurons through backpropagation. Backpropagation is activated based on the stochastic gradient descent which minimizes the loss function of the respective outputs of the Multi Layer Perceptron through the training process.

#### 3.3.4. Error Analysis

For each prediction, i.e., consisting of $N_{p}$ different generated random walks labeled with *i* for each different Hurst exponent *h*, we calculated the mean and the standard deviation as   

(7) $$\hat{X}\left(h\right)=\frac{1}{N_{p}}\sum_{i=1}^{N_{p}}{\hat{X}}_{i}\left(h\right)\;,\;\sigma \left(h\right)=\sqrt{\frac{1}{N_{p}}\sum_{i=1}^{N_{p}}{\left({\hat{X}}_{i}\left(h\right)-\hat{X}\left(h\right)\right)}^{2}}\;,$$

where ${\hat{X}}_{i}\left(h\right)$ is a single observation, $\hat{X}\left(h\right)$ is the averaged observation for a single Hurst exponent, $\sigma \left(h\right)$ is the corresponding standard deviation and $N_{p}$ is the number of different random walks for each Hurst exponent.

Next, to compare it to the ground truth, we calculated the root-mean-square error (RMSE) as   

(8) $$E_{RMSE}\;=\;{\left(\frac{1}{N_{h}}\sum_{h=1}^{N_{h}}{\left[\hat{X}\left(h\right)\;-\;X\left(h\right)\right]}^{2}\right)}^{\frac{1}{2}}\;,$$

where $X\left(h\right)$ is the ground truth of the Hurst exponent for each random walk and $N_{h}$ is the number of validation data points, i.e., different tested Hurst exponents and, consequently, random walks. Using error propagation, the corresponding error of the root-mean-square error is found as

(9) $$\Delta E_{RMSE}=\sqrt{{\left(\frac{\partial E_{RMSE}}{\partial \hat{X}\left(1\right)}\right)}^{2}\sigma^{2}\left(1\right)+{\left(\frac{\partial E_{RMSE}}{\partial \hat{X}\left(2\right)}\right)}^{2}\sigma^{2}\left(2\right)+\;\cdots }\;,$$

thus yielding:   

(10) $$\Delta E_{RMSE}\;=\;\sqrt{\frac{\sum_{h=1}^{N_{h}}{\left[\hat{X}\left(h\right)\;-\;X\left(h\right)\right]}^{2}\;\sigma^{2}\left(h\right)}{\left(N_{h}\right)*\sum_{h=1}^{N_{h}}{\left[\hat{X}\left(h\right)\;-\;X\left(h\right)\right]}^{2}}}\;.$$

## 4. Machine Learning Training/Validation

In this section, we present a comprehensive overview of our experimental approach, detailing the generation of training data, the application of machine learning algorithms, and the evaluation of their accuracy in estimating the Hurst exponent for time series data.

Our experimental design encompasses several key steps, beginning with the generation of training data, which includes both fractional Brownian and fractional Lévy motion. We incorporate varying random walk lengths and utilize the known or defined scaling parameter as ground truth. Subsequently, we train a diverse array of machine learning algorithms using these datasets, encompassing all three scenarios: fractional Brownian motion, fractional Lévy motion, and a combination of both.

Upon completing the training phase, we evaluate the performance of each trained algorithm using newly generated random walks—ensuring that these walks were not part of the training data. To guarantee the reliability of our findings, we provide a sufficiently large statistical sample and the corresponding variability for each algorithm, encompassing each type of data and the associated Hurst exponents.

Lastly, we compare our results with well-established classical algorithms from various software packages designed to estimate the Hurst exponent, or scaling parameter, of the data under investigation. This comparison enables us to assess the effectiveness of our machine learning-based approach relative to traditional methods.

We provide all program code, an application to estimate the Hurst exponent using our best models and all data in the author’s GitHub repository (https://github.com/Raubkatz/ML_Hurst_Estimation) (accessed on 13 December 2023) [31].

### 4.1. Training Data

In generating our training data, we utilized fractional Brownian and fractional Lévy motions, which we initialized with varying parameters. For the fractional Brownian motion (described in Section 3.1), we adjusted the Hurst exponent to be within the range of $H\in \left[0.001,0.999\right]$. For the fractional Lévy motion, we varied the Lévy index, α, and the scaling parameter, here referred to as H, as it exhibits similarities to the Hurst exponent, though not entirely the same. In this case, $\alpha \in \left[0.1,1.999\right]$ and $H\in \left[0.001,0.999\right]$. The Lévy index, α, serves as an essential parameter in understanding and controlling the heavy-tailedness of the fractional Lévy motion, shaping the properties of the resulting stochastic process.

For both fractional Brownian and fractional Lévy motions, we generated 50,000 random walks, each consisting of 100,000 equidistant data points. We randomly selected excerpts from each of these random walks with a probability of p=0.15 to be used as training samples. These sampled excerpts are a time series of different lengths—i.e., 10, 25, 50, 100—which then served, normalized to the unit interval [0,1], directly as the input for our machine learning algorithms. These samples were then saved alongside their corresponding Hurst/scaling exponent, which serves as the ground truth and, consequently, the value to be predicted. Consequently, we obtained three datasets for each signal length: one containing only fractional Brownian motion, one with only fractional Lévy motion, and one comprising both types of motion. Since we randomly selected excerpts from each generated random walk, the number of samples slightly fluctuated, resulting in approximately 743,000 unmixed random walk data points, i.e., consisting of either fractional Brownian or Lévy motion, and approximately double the number of samples in the combined dataset. To ensure the reproducibility of our experiments, we provide the complete datasets in our GitHub repository (https://github.com/Raubkatz/ML_Hurst_Estimation) (accessed on 13 December 2023) [31].

### 4.2. Training the Machine Learning Models

In order to train our models, we leveraged the well-established scikit-learn library [30], employing a 5-fold cross-validation along with an $r^{2}$ score to indicate good or bad fits. We combined this with a random parameter search, specifically the RandomizedSearchCV, with 40 iterations. A detailed discussion of all hyperparameters for each model and the training process is not provided here, as we have made the complete program code, as well as the trained models, available in the linked Github repository. The cross-validation scores for all models can be found in Table 1. We conclude from our cross-validation for training the various machine learning models that CatBoost is the preferred algorithm because it performs overall well and rather stable. Overall, the two advanced Boosting-Regressors, LightGBM, and CatBoost, delivered the best performance.

### 4.3. Validating the Trained Models

This section outlines the methodology used to evaluate all the trained models. To make sure the validation procedure did not include parts of the training data, we created all random walks for validation separately. The validation process was executed as follows:

First, we defined a range of equidistant Hurst exponents for model evaluation. This discrete list of Hurst/scaling exponents includes 0.025,0.05,0.075,0.1,…,0.975. Moreover, we selected three different values for the Lévy index of the included fractional Lévy motion, i.e., α∈0.5,1.0,1.5. Next, for each of these Hurst exponents, we generated a random walk with a length of 200,000 data points. From these random walks, we extracted 1000 excerpts of varying lengths that matched the model inputs, i.e., 10,25,50,100, along with a window length of 200 and 350 data points.

To estimate the Hurst exponent for the window sizes of 200 and 250, we calculated sliding windows of 100 data points over the 200 and 350 data points and then averaged the results, as we did not train models with inputs larger than 100 consecutive data points. These two input window lengths are chosen in alignment with the findings of this article, specifically, the experiments on financial time series data from Section 5, and the work done by [32].

We evaluated all techniques for estimating the scaling exponent of a time series data by calculating an RMSE showing the difference from the ground truth of the sample, i.e., how much the employed technique to estimate the scaling exponent of a random walk is off in the “Hurst” scale. Additionally, we obtained a corresponding error to characterize the variability of these results, which is discussed in detail in Section 3.3.4.

The results of these experiments are shown in the following tables. First, we tested our trained models on only fractional Brownian motions (Table 2 and Table 3). For the “classical” algorithms, we found that DFA outperforms everything else. However, DFA suffers from a large variability compared to other algorithms and trained machine learning models for small window lengths. For the trained machine learning models, we found that CatBoost, trained on data obtained only from fractional Brownian motions, performs best for all window lengths except for 10 input data points where LightGBM performs best. Also, except for a window length of 10, the best-trained machine learning models outperform all tested “classical” algorithms.

Next, for fractional Lévy motion with α=0.5 (Table 4 and Table 5), we observe that the R/S analysis from the nolds Python package performs best for window lengths up to 50 data points. Beyond this threshold, the R/S analysis from the Python package hurst performs best. We conclude from these results that DFA, despite its reputation, is a very unreliable tool to estimate the scaling exponent of non-fractional-Brownian-processes. For the trained machine learning models, we note that LightGBM and CatBoost perform best for input windows of below 100 data points, whereas above this threshold, the best results come from the multi-layer perceptron. For these datasets, the trained machine learning models outperformed all classical algorithms. Furthermore, we obtained the best results from machine learning models trained only on fractional Lévy time series data.

Next, for fractional Lévy motion with α=1.0 (Table 6 and Table 7), DFA performs best for the window lengths of below 100 data points, and the simplified R/S analysis from the Python package hurst performs best above this threshold for the classical algorithms. What is interesting here is that, except for an input window of 25 data points where the MLP performs best, CatBoost outperforms all of the other machine learning models. Furthermore, the simplified R/S analysis outperforms the machine learning models for a length of 200 and 350 data points, whereas for other input lengths, trained machine learning models perform best. Interestingly, for these random processes, the machine learning models trained with fractional Brownian motion perform best, contrary to fractional Lévy motion with α=0.5.

For the fractional Lévy motion validation dataset with α=1.5 (Table 8 and Table 9), we observe that DFA performs best for samples with lengths below 100 data points, and the R/S analysis from the Python package hurst performs best for the remaining window lengths for the classical estimation methods. For the machine learning models, we note that LightGBM performs best for window lengths of 10 and 25, whereas MLP performs best for the remaining sample sizes. Surprisingly, we obtain the best results for the trained machine learning models only for models trained with fractional Brownian motions, which is not what one would expect. This might be due to the drastic differences of fractional Lévy motion with varying α. Still, the trained machine learning models outperform all classical algorithms to estimate the scaling parameter of this time series data. However, the MLP algorithms trained on both fractional Brownian and Lévy motions are very close to the ones trained on only fractional Brownian motions for a sample size of 100, 200, and 350 consecutive data points.

In conclusion, trained machine learning models generally outperformed classical algorithms in estimating the scaling exponent across all types of data. Machine learning models trained on fractional Brownian motion showed unexpected advantages even when applied to fractional Lévy motion data.

#### 4.3.1. Fractional Brownian Motion

**Figure 1:**
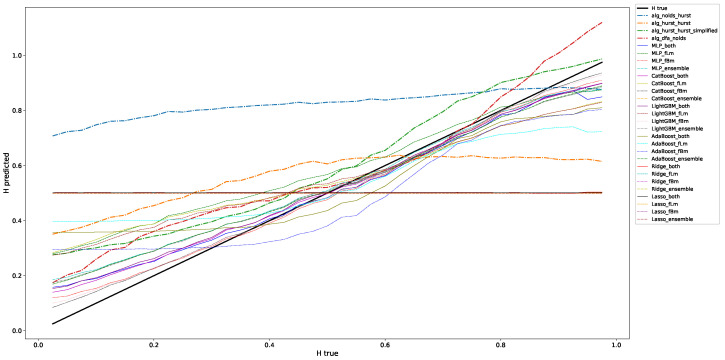
This figure presents a correlation plot illustrating the relationship between predicted and actual values in estimating the Hurst exponent for fractional Brownian motion data. The horizontal axis represents the true Hurst values, while the vertical axis shows the predicted values by various algorithms. These are the results for a window length of 100 data points from Table 2 and Table 3.

This section contains the results for the case of the classic fractional Brownian motion. We depicted the results from Table 2 and Table 3 for a window length of 100 data points in Figure 1. This figure depicts what the information that we condensed into the following tables means: We check for a range of Hurst exponents on how well we can approximate the ground truth, which in this Figure is the black line from (0.0/0.0) to (1.0,1.0). The RMSEs and corresponding variabilities refer to the distance to this ground truth. This figure shows that, given that we interpret it visually, the machine learning algorithms, except for Lasso and Ridge, do a good job in predicting the correct Hurst value compared to the classical algorithms, such that they are close to the ground truth. Further, the RMSE-errors given in the regarded, and in all following tables (Table 4, Table 5, Table 6, Table 7, Table 8 and Table 9), indicate the variability of the employed technique to predict the correct scaling exponent. This means that an algorithm that, on average, has a low RMSE but suffers from a large variability provides highly fluctuating results on predicting scaling exponents and/or that it could be very off for large or small scaling exponents, just as shown for, e.g., alg_hurst_hurst in Figure 1.

**Table 2:** RMSE for each of the non-machine-learning algorithms for 1000 analyzed fractional Brownian motions for varying input window sizes. We highlight the lowest errors for each window size by using a bold font type.

Window Length	Alg. Nolds Hurst	Alg. Nolds DFA	Alg. Hurst Hurst	Alg. Hurst Hurst Simplified
10	0.2916 ± 0.01531	**0.0751 ± 0.24194**	-	-
25	0.3302 ± 0.01983	**0.09547 ± 0.04252**	-	-
50	0.36642 ± 0.01355	**0.10151 ± 0.02645**	-	-
100	0.40063 ± 0.01175	**0.10392 ± 0.019**	0.21317 ± 0.01494	0.114 ± 0.01291
200	0.41373 ± 0.00816	**0.06301 ± 0.01224**	0.19125 ± 0.01136	0.10718 ± 0.00966
350	0.42249 ± 0.00697	**0.04495 ± 0.0069**	0.18045 ± 0.01012	0.10363 ± 0.00875

**Table 3:** RMSE for the trained machine learning models for 1000 analyzed fractional Brownian motions for varying input window sizes. We highlight the lowest errors for each window size by using a bold font type.

Window Length	Ridge fBm	Ridge fLm	Ridge Both
10	0.28137 ± 0.00024	0.28134 ± 0.00071	0.28136 ± 0.00045
25	0.28157 ± 0.00033	0.28144 ± 0.0004	0.28161 ± 0.00022
50	0.28125 ± 0.00051	0.28137 ± 0.00056	0.28124 ± 0.00043
100	0.28144 ± 0.00088	0.28123 ± 0.00122	0.28138 ± 0.00089
200	0.28126 ± 0.00011	0.28131 ± 0.00071	0.28122 ± 0.00053
350	0.2814 ± 0.00012	0.28132 ± 0.0005	0.28137 ± 0.00038
**Window Length**	**Lasso fBm**	**Lasso fLm**	**Lasso both**
10	0.28137 ± 0.0	0.28137 ± 0.0	0.28137 ± 0.0
25	0.28137 ± 0.0	0.28137 ± 0.0	0.28137 ± 0.0
50	0.28137 ± 0.0	0.28137 ± 0.0	0.28137 ± 0.0
100	0.28137 ± 0.0	0.28137 ± 0.0	0.28137 ± 0.0
200	0.28137 ± 0.0	0.28137 ± 0.0	0.28137 ± 0.0
350	0.28137 ± 0.0	0.28137 ± 0.0	0.28137 ± 0.0
**Window Length**	**AdaBoost fBm**	**AdaBoost fLm**	**AdaBoost Both**
10	0.22244 ± 0.01316	0.2262 ± 0.013	0.21941 ± 0.01388
25	0.16691 ± 0.01471	0.19494 ± 0.00965	0.17723 ± 0.01186
50	0.14028 ± 0.01172	0.17266 ± 0.00652	0.14778 ± 0.00961
100	0.11962 ± 0.01022	0.16265 ± 0.00337	0.13449 ± 0.00574
200	0.12036 ± 0.00652	0.16359 ± 0.00161	0.1346 ± 0.00317
350	0.12079 ± 0.00461	0.16256 ± 0.00117	0.13533 ± 0.00224
**Window Length**	**CatBoost fBm**	**CatBoost fLm**	**CatBoost Both**
10	0.16014 ± 0.02044	0.21121 ± 0.01504	0.1782 ± 0.01751
25	**0.08767 ± 0.01857**	0.1851 ± 0.01472	0.11832 ± 0.01546
50	**0.05087 ± 0.01546**	0.16244 ± 0.01481	0.07659 ± 0.0142
100	**0.02484 ± 0.01112**	0.13197 ± 0.01461	0.04776 ± 0.01179
200	**0.026 ± 0.00839**	0.13222 ± 0.0105	0.04831 ± 0.00879
350	**0.0257 ± 0.00625**	0.13197 ± 0.00765	0.0479 ± 0.00651
**Window Length**	**LightGBM fBm**	**LightGBM fLm**	**LightGBM Both**
10	**0.16011 ± 0.02052**	0.21073 ± 0.01527	0.17831 ± 0.01753
25	0.09248 ± 0.01834	0.18331 ± 0.01444	0.12301 ± 0.01514
50	0.05323 ± 0.01538	0.15624 ± 0.01414	0.08313 ± 0.01379
100	0.02802 ± 0.01073	0.12563 ± 0.01423	0.05224 ± 0.01161
200	0.02904 ± 0.00821	0.12482 ± 0.01025	0.05178 ± 0.00821
350	0.02863 ± 0.00605	0.12465 ± 0.0075	0.05151 ± 0.00608
**Window Length**	**MLP fBm**	**MLP fLm**	**MLP Both**
10	0.1656 ± 0.02182	0.21432 ± 0.01481	0.17702 ± 0.01761
25	0.09959 ± 0.01788	0.17948 ± 0.01469	0.1326 ± 0.01475
50	0.06085 ± 0.01463	0.14635 ± 0.01535	0.08806 ± 0.01467
100	0.03473 ± 0.00988	0.12899 ± 0.01635	0.05588 ± 0.01101
200	0.03578 ± 0.00755	0.12915 ± 0.01183	0.05784 ± 0.00798
350	0.03586 ± 0.00541	0.12858 ± 0.00873	0.05516 ± 0.00615

#### 4.3.2. Fractional Lévy Motion, α = 0.5

This section contains the results for the case of fractional Lévy motion with α=0.5.

**Table 4:** RMSE for each of the non-machine learning algorithms for 1000 analyzed fractional Brownian motions for varying input window sizes. We highlight the lowest errors for each window size by using a bold font type.

Window Length	Alg. Nolds Hurst	Alg. Nolds DFA	Alg. Hurst Hurst	Alg. Hurst Hurst Simplified
10	**0.28517 ± 0.01622**	0.42035 ± 0.2706	-	-
25	**0.26316 ± 0.02612**	0.47115 ± 0.0471	-	-
50	**0.26357 ± 0.01986**	0.45586 ± 0.03011	-	-
100	0.28779 ± 0.01684	0.45104 ± 0.02172	**0.21913 ± 0.01621**	0.24127 ± 0.01786
200	0.31121 ± 0.01129	0.46071 ± 0.01456	**0.22713 ± 0.01267**	0.24231 ± 0.01428
350	0.33902 ± 0.00942	0.44371 ± 0.01141	**0.23226 ± 0.01066**	0.24178 ± 0.01233

**Table 5:** RMSE for the trained machine learning models for 1000 analyzed fractional Brownian motions for varying input window sizes. We highlight the lowest errors for each window size by using a bold font type.

Window Length	Ridge fBm	Ridge fLm	Ridge both
10	0.28139 ± 0.0003	0.28116 ± 0.00077	0.28123 ± 0.00053
25	0.28133 ± 0.00053	0.28131 ± 0.00052	0.28135 ± 0.00032
50	0.28134 ± 0.00085	0.28135 ± 0.00079	0.28131 ± 0.00057
100	0.28141 ± 0.0015	0.28124 ± 0.00148	0.28122 ± 0.00111
200	0.28136 ± 8 $\times 10^{-5}$	0.28122 ± 0.00079	0.28121 ± 0.00058
350	0.28133 ± 6 $\times 10^{-5}$	0.28109 ± 0.00055	0.28109 ± 0.00041
**Window Length**	**Lasso fBm**	**Lasso fLm**	**Lasso Both**
10	0.28137 ± 0.0	0.28137 ± 0.0	0.28137 ± 0.0
25	0.28137 ± 0.0	0.28137 ± 0.0	0.28137 ± 0.0
50	0.28137 ± 0.0	0.28137 ± 0.0	0.28137 ± 0.0
100	0.28137 ± 0.0	0.28137 ± 0.0	0.28137 ± 0.0
200	0.28137 ± 0.0	0.28137 ± 0.0	0.28137 ± 0.0
350	0.28137 ± 0.0	0.28137 ± 0.0	0.28137 ± 0.0
**Window Length**	**AdaBoost fBm**	**AdaBoost fLm**	**AdaBoost Both**
10	0.28501 ± 0.00965	0.27944 ± 0.00736	0.28093 ± 0.0092
25	0.2994 ± 0.0117	0.27436 ± 0.00838	0.2817 ± 0.01012
50	0.30762 ± 0.01202	0.26953 ± 0.00996	0.28504 ± 0.01142
100	0.32061 ± 0.01262	0.26418 ± 0.01149	0.28198 ± 0.01247
200	0.3201 ± 0.00709	0.26357 ± 0.00652	0.28126 ± 0.00638
350	0.31737 ± 0.00536	0.26173 ± 0.0047	0.27918 ± 0.00468
**Window Length**	**CatBoost fBm**	**CatBoost fLm**	**CatBoost Both**
10	0.29105 ± 0.01685	0.26013 ± 0.01451	0.27373 ± 0.01506
25	0.31954 ± 0.02005	**0.22098 ± 0.01589**	0.25413 ± 0.01666
50	0.32883 ± 0.01971	**0.19311 ± 0.01505**	0.22873 ± 0.01656
100	0.32947 ± 0.01705	0.18257 ± 0.01354	0.21623 ± 0.01513
200	0.3283 ± 0.01265	0.18206 ± 0.00866	0.21506 ± 0.01052
350	0.3265 ± 0.00934	0.18128 ± 0.00607	0.21404 ± 0.00751
**Window Length**	**LightGBM fBm**	**LightGBM fLm**	**LightGBM Both**
10	0.28999 ± 0.01713	**0.25899 ± 0.01505**	0.2733 ± 0.01536
25	0.31583 ± 0.02002	0.22418 ± 0.016	0.25836 ± 0.01676
50	0.32294 ± 0.02003	0.1996 ± 0.01483	0.23572 ± 0.01697
100	0.30969 ± 0.01737	0.18867 ± 0.01346	0.2209 ± 0.01607
200	0.30965 ± 0.0128	0.1884 ± 0.00876	0.22123 ± 0.01096
350	0.30776 ± 0.00946	0.18759 ± 0.00615	0.22019 ± 0.00783
**Window Length**	**MLP fBm**	**MLP fLm**	**MLP Both**
10	0.30555 ± 0.01746	0.26038 ± 0.01426	0.27412 ± 0.0149
25	0.33234 ± 0.01994	0.23591 ± 0.01552	0.25557 ± 0.01708
50	0.33114 ± 0.02127	0.20713 ± 0.01606	0.23219 ± 0.01847
100	0.29554 ± 0.01763	**0.18192 ± 0.01455**	0.23114 ± 0.01596
200	0.29431 ± 0.01297	**0.18123 ± 0.00948**	0.2301 ± 0.01107
350	0.29222 ± 0.00954	**0.18008 ± 0.00668**	0.22919 ± 0.00788

#### 4.3.3. Fractional Lévy Motion, α = 1.0

This section contains the results for the case of fractional Lévy motion with α=1.0.

**Table 6:** RMSE for each of the non-machine learning algorithms for 1000 analyzed fractional Brownian motions for varying input window sizes. We highlight the lowest errors for each window size by using a bold font type.

Window Length	Alg. Nolds Hurst	Alg. Nolds DFA	Alg. Hurst Hurst	Alg. Hurst Hurst Simplified
10	0.28169 ± 0.01582	**0.14867 ± 0.24577**	-	-
25	0.26134 ± 0.02507	**0.16672 ± 0.04256**	-	-
50	0.29846 ± 0.01826	**0.15482 ± 0.02765**	-	-
100	0.34383 ± 0.01472	0.15946 ± 0.01826	0.11358 ± 0.01295	**0.09771 ± 0.0146**
200	0.37387 ± 0.00979	0.15343 ± 0.01281	0.09689 ± 0.00983	**0.09633 ± 0.01395**
350	0.40277 ± 0.00817	0.14777 ± 0.00979	0.08666 ± 0.0082	**0.08423 ± 0.01146**

**Table 7:** RMSE for the trained machine learning models for 1000 analyzed fractional Brownian motions for varying input window sizes. We highlight the lowest errors for each window size by using a bold font type.

Window Length	Ridge fBm	Ridge fLm	Ridge Both
10	0.28117 ± 0.00027	0.28126 ± 0.00072	0.28145 ± 0.00048
25	0.28131 ± 0.00043	0.28134 ± 0.00046	0.2813 ± 0.00027
50	0.2812 ± 0.00069	0.28136 ± 0.00067	0.28124 ± 0.00051
100	0.28138 ± 0.00121	0.28139 ± 0.00133	0.28141 ± 0.00101
200	0.28127 ± 0.00013	0.28133 ± 0.00081	0.28124 ± 0.0006
350	0.28129 ± 0.00011	0.28135 ± 0.00052	0.28129 ± 0.00039
**Window Length**	**Lasso fBm**	**Lasso fLm**	**Lasso Both**
10	0.28137 ± 0.0	0.28137 ± 0.0	0.28137 ± 0.0
25	0.28137 ± 0.0	0.28137 ± 0.0	0.28137 ± 0.0
50	0.28137 ± 0.0	0.28137 ± 0.0	0.28137 ± 0.0
100	0.28137 ± 0.0	0.28137 ± 0.0	0.28137 ± 0.0
200	0.28137 ± 0.0	0.28137 ± 0.0	0.28137 ± 0.0
350	0.28137 ± 0.0	0.28137 ± 0.0	0.28137 ± 0.0
**Window Length**	**AdaBoost fBm**	**AdaBoost fLm**	**AdaBoost Both**
10	0.22433 ± 0.0129	0.22269 ± 0.01196	0.21992 ± 0.01335
25	0.18802 ± 0.01813	0.20001 ± 0.01125	0.19137 ± 0.01486
50	0.18038 ± 0.0187	0.19031 ± 0.0111	0.17882 ± 0.01649
100	0.17227 ± 0.01964	0.1828 ± 0.00976	0.17062 ± 0.01527
200	0.19107 ± 0.01682	0.19405 ± 0.00888	0.18627 ± 0.01331
350	0.1832 ± 0.01297	0.18972 ± 0.00662	0.17945 ± 0.01015
**Window Length**	**CatBoost fBm**	**CatBoost fLm**	**CatBoost Both**
10	**0.16251 ± 0.0228**	0.1735 ± 0.0155	0.16663 ± 0.01876
25	0.10053 ± 0.02435	0.12608 ± 0.01514	0.11452 ± 0.01884
50	**0.08678 ± 0.0223**	0.10853 ± 0.0148	0.09634 ± 0.01796
100	**0.0805 ± 0.01776**	0.09093 ± 0.0143	0.08429 ± 0.01576
200	**0.09697 ± 0.01578**	0.11339 ± 0.01109	0.10593 ± 0.01341
350	**0.08847 ± 0.01262**	0.10591 ± 0.009	0.09767 ± 0.01095
**Window Length**	**LightGBM fBm**	**LightGBM fLm**	**LightGBM Both**
10	0.16298 ± 0.02281	0.17226 ± 0.0156	0.1669 ± 0.01887
25	0.10358 ± 0.02465	0.12709 ± 0.01515	0.11801 ± 0.01879
50	0.08901 ± 0.02287	0.11128 ± 0.01457	0.0991 ± 0.01792
100	0.08219 ± 0.01884	0.09293 ± 0.01406	0.08662 ± 0.01601
200	0.10263 ± 0.01659	0.11551 ± 0.01118	0.10916 ± 0.01362
350	0.09302 ± 0.01335	0.10787 ± 0.00902	0.10049 ± 0.01107
**Window Length**	**MLP fBm**	**MLP fLm**	**MLP Both**
10	0.16992 ± 0.02453	0.17416 ± 0.01483	0.1626 ± 0.01924
25	**0.0989 ± 0.02508**	0.12536 ± 0.01521	0.11192 ± 0.01802
50	0.087 ± 0.02189	0.10809 ± 0.01586	0.09365 ± 0.01821
100	0.08741 ± 0.01875	0.09523 ± 0.01581	0.09705 ± 0.01728
200	0.10958 ± 0.01624	0.11189 ± 0.01236	0.12114 ± 0.01475
350	0.10021 ± 0.01313	0.10521 ± 0.00936	0.11197 ± 0.01189

#### 4.3.4. Fractional Lévy Motion, α = 1.5

This section contains the results for the case of fractional Lévy motion with α=1.5.

**Table 8:** RMSE for each of the non-machine learning algorithms for 1000 analyzed fractional Brownian motions for varying input window sizes. We highlight the lowest errors for each window size by using a bold font type.

Window Length	Alg. Nolds Hurst	Alg. Nolds DFA	Alg. Hurst Hurst	Alg. Hurst Hurst Simplified
10	0.27084 ± 0.01484	**0.23363 ± 0.21841**	-	-
25	0.28817 ± 0.02327	**0.23679 ± 0.0342**	-	-
50	0.33359 ± 0.01618	**0.25416 ± 0.01981**	-	-
100	0.3782 ± 0.01311	0.25933 ± 0.01619	0.21506 ± 0.01824	**0.21175 ± 0.02937**
200	0.40683 ± 0.00869	0.23166 ± 0.01267	**0.20716 ± 0.01488**	0.20741 ± 0.02327
350	0.43147 ± 0.00722	0.21754 ± 0.01074	**0.20099 ± 0.01288**	0.20322 ± 0.02032

**Table 9:** RMSE for the trained machine learning models for 1000 analyzed fractional Brownian motions for varying input window sizes. We highlight the lowest errors for each window size by using a bold font type.

Window Length	Ridge fBm	Ridge fLm	Ridge Both
10	0.28124 ± 0.00023	0.2811 ± 0.00061	0.28131 ± 0.00042
25	0.28142 ± 0.0004	0.2813 ± 0.0004	0.28145 ± 0.00026
50	0.28148 ± 0.00062	0.28155 ± 0.00058	0.28168 ± 0.00045
100	0.28118 ± 0.00108	0.28203 ± 0.00117	0.28168 ± 0.00088
200	0.28143 ± 0.00013	0.28202 ± 0.00069	0.28194 ± 0.00052
350	0.28158 ± 9 $\times 10^{-5}$	0.28202 ± 0.00046	0.28208 ± 0.00034
**Window Length**	**Lasso fBm**	**Lasso fLm**	**Lasso Both**
10	0.28137 ± 0.0	0.28137 ± 0.0	0.28137 ± 0.0
25	0.28137 ± 0.0	0.28137 ± 0.0	0.28137 ± 0.0
50	0.28137 ± 0.0	0.28137 ± 0.0	0.28137 ± 0.0
100	0.28137 ± 0.0	0.28137 ± 0.0	0.28137 ± 0.0
200	0.28137 ± 0.0	0.28137 ± 0.0	0.28137 ± 0.0
350	0.28137 ± 0.0	0.28137 ± 0.0	0.28137 ± 0.0
**Window Length**	**AdaBoost fBm**	**AdaBoost fLm**	**AdaBoost Both**
10	0.17954 ± 0.00739	0.17841 ± 0.00603	0.17501 ± 0.0072
25	0.13308 ± 0.00729	0.16829 ± 0.00594	0.15288 ± 0.00664
50	0.11901 ± 0.00597	0.16298 ± 0.00575	0.13967 ± 0.00629
100	0.10492 ± 0.00603	0.16836 ± 0.0062	0.13647 ± 0.00699
200	0.10557 ± 0.00475	0.1677 ± 0.00428	0.13728 ± 0.00471
350	0.10388 ± 0.00248	0.16646 ± 0.00301	0.13614 ± 0.00312
**Window Length**	**CatBoost fBm**	**CatBoost fLm**	**CatBoost Both**
10	0.12585 ± 0.01533	0.13493 ± 0.01381	0.12543 ± 0.01354
25	0.09966 ± 0.02066	0.11572 ± 0.01648	0.10284 ± 0.01716
50	0.09861 ± 0.01676	0.10646 ± 0.0161	0.09885 ± 0.0167
100	0.11132 ± 0.01521	0.10493 ± 0.01517	0.10261 ± 0.01542
200	0.10422 ± 0.01406	0.10233 ± 0.01111	0.09901 ± 0.01235
350	0.10423 ± 0.01236	0.10089 ± 0.00847	0.09761 ± 0.00979
**Window Length**	**LightGBM fBm**	**LightGBM fLm**	**LightGBM Both**
10	**0.12474 ± 0.01531**	0.13449 ± 0.01421	0.12653 ± 0.01365
25	**0.09164 ± 0.02074**	0.11827 ± 0.01639	0.10419 ± 0.01662
50	0.09421 ± 0.01709	0.1128 ± 0.01605	0.1014 ± 0.01649
100	0.10036 ± 0.01603	0.10926 ± 0.0156	0.10413 ± 0.01581
200	0.09374 ± 0.0143	0.10574 ± 0.01141	0.09917 ± 0.01238
350	0.0929 ± 0.01228	0.10395 ± 0.00866	0.09765 ± 0.00978
**Window Length**	**MLP fBm**	**MLP fLm**	**MLP Both**
10	0.10349 ± 0.01432	0.13397 ± 0.01323	0.12935 ± 0.01348
25	0.09597 ± 0.02127	0.11178 ± 0.01572	0.11672 ± 0.01739
50	**0.08883 ± 0.01777**	0.11371 ± 0.0167	0.12015 ± 0.018
100	**0.09425 ± 0.01593**	0.13946 ± 0.01676	0.09106 ± 0.01621
200	**0.08884 ± 0.01365**	0.13815 ± 0.01241	0.0876 ± 0.01283
350	**0.08686 ± 0.01138**	0.1367 ± 0.00935	0.08641 ± 0.01017

## 5. Finance Experiments

The dynamics of stock market analysis have been gaining considerable attention from both researchers and practitioners for a long time. This interest is primarily directed towards understanding the underlying patterns and structures present in financial time series data. A popular approach to characterizing stock market behavior involves estimating the Hurst or scaling exponent. This measure indicates the long-range dependence and persistence in time series data [1,5]. This concept has been implemented in more recent studies, thereby enhancing our understanding of stock market dynamics. Previous research by [32], for instance, utilized Detrended Fluctuation Analysis (DFA) to estimate the Hurst or scaling exponent in the Dow Jones and S&P 500 daily indices. This approach unveiled significant insights into the time-varying nature of these markets. Additionally, DFA, and/or the estimation of the scaling exponent of a time series data can also be applied to analyze the volatility of stock market data [33].

Building on this foundational work, the present study aims to expand upon the analysis by incorporating a machine learning approach to estimate the Hurst or scaling exponent for financial time series data. This introduces a more scalable and robust method for studying the multifractal structure of stock market data. By leveraging the power of machine learning algorithms, we are able to reliably employ smaller sliding windows for analysis, thereby capturing more nuanced and detailed variations in the Hurst exponent over time.

**Figure 2:**
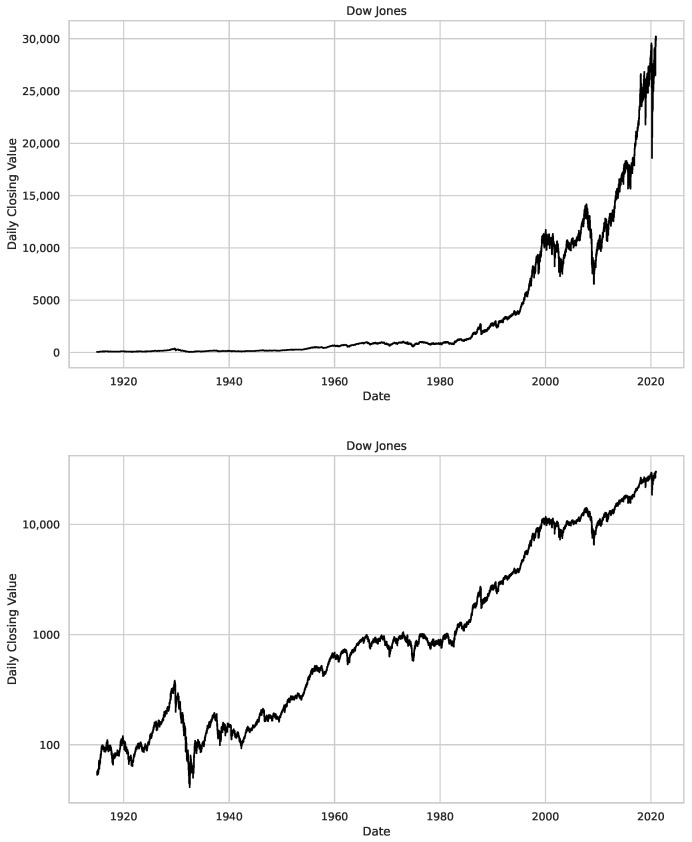

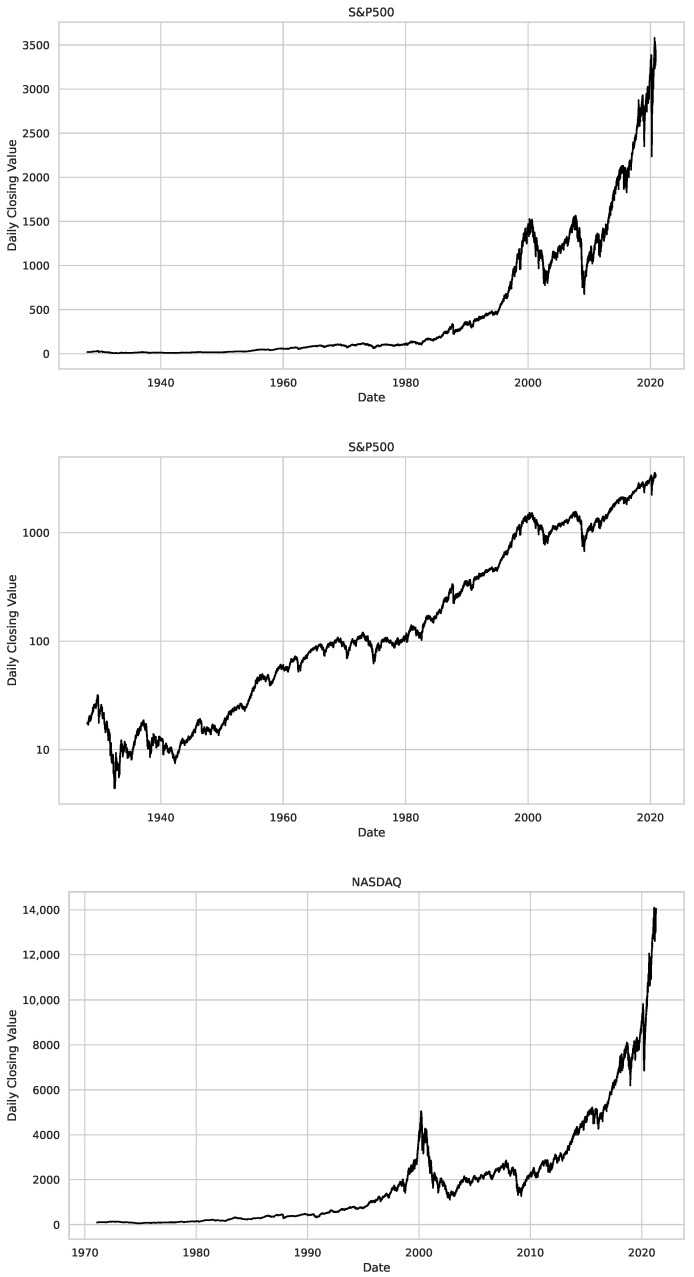

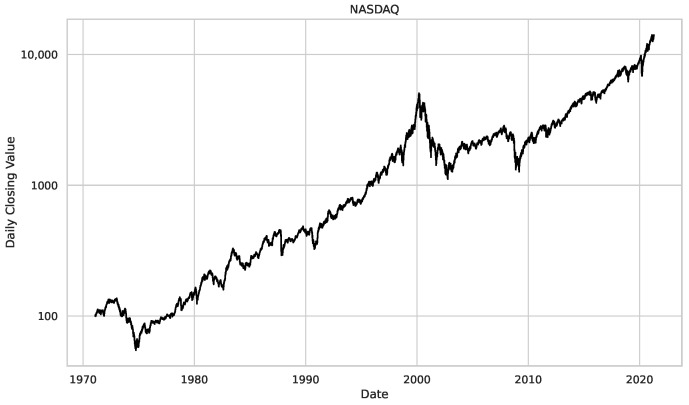
Time series plots for the daily closing values of the assets used in our study, i.e., Dow Jones, S&P 500 and NASDAQ. On the left side, we see regular plots, and on the right side, the y-axis is in a logarithmic scale, illustrating the relative changes in the value of the assets. We studied the Dow Jones from 12 December 1914 to 15 December 2020, S&P500 goes from 30 December 1927 to the 4 November 2020 and for the NASDAQ the studied period starts on the 5 February 1972 and ends on the 16 April 2021.

We perform our analysis in a manner similar to the work by [32], to evaluate the capabilities of the developed method. However, our study expands the scope of this analysis by incorporating daily closed values from not only the Dow Jones and S&P 500 but also NASDAQ. The used daily closing value time series data are depicted and described in Figure 2. It is important to mention here that, unlike the data from [32], we use only daily closed values due to data availability, not the mean between open and closed values.

In this section, we provide a brief discussion of related literature on stochastic processes and financial data. We then employ a slightly modified version of R/S analysis to determine the stochastic process the data under study is related to, i.e., fractional Brownian or fractional Lévy motion, and thus adjust our model and algorithm selection accordingly. Following this, we apply traditional algorithms and trained machine learning models to estimate the scaling exponent of the data under study in a sliding window over several decades to show the changes in fractality and scaling behavior. Finally, we conclude our findings and compare them to the work undertaken by [32], and analyze how the different methods employed to estimate the scaling exponent of financial time series data correlate with each other.

### 5.1. The Scaling Exponent of Financial Data

The study of financial data in the context of fractional Brownian motion (fBm) and fractional Lévy motion (fLm) has revealed a multifractal nature rather than a simple monofractal one [16,34,35,36,37]. Both of these processes allow for dependency in increments, characterized by the Hurst parameter H. It is understood that financial time series often exhibit long-range dependence and volatility clustering, which can be captured by fBm when H>0.5.

However, when comparing the behavior of fractional Brownian motions to that of financial markets, Ivanova et al. [37] found that the behavior of financial markets, e.g., Dow Jones, is rather multifractal than that of a monofractal Brownian motion. In terms of the Hurst exponent for multifractal data, one observes fluctuations even on data where the Hurst exponent should be constant, i.e., monofractal data [38], hinting that the employed algorithms might not be capable of reliably determining the scaling exponent of stochastic data on small intervals. This observation aligns with our findings from previous sections, i.e., the Hurst exponent looks locally, sometimes very different from the ground truth. This serves as further evidence that the estimation of the Hurst exponent needs to be scaled down to smaller intervals because of the multifractality of stochastic and financial data. Another factor to consider is that stochastic data (e.g., fractional Brownian motion) with a given Hurst exponent, e.g., H=0.3 (heavily fluctuating), might look on small intervals like stochastic data with an increased Hurst exponent, e.g., H=0.7, if the observed data locally exhibits the behavior of a mostly straight line, which sometimes happens due to the probabilistic nature of these processes.

Interestingly, when examining the properties of financial data in relation to fBm and fLm, it appears that financial data, such as European option data, align more closely with fLm [23]. This is further investigated by the work of Barunik et al. [16]. In their study, they observed an interesting phenomenon where an apparent increase in multifractality was measured in time series generated from shuffled returns, even when all time-correlations were destroyed and only return distributions were conserved. Their investigation into this phenomenon included the simulation of time series via the Markov switching multifractal model, autoregressive fractionally integrated moving average processes with stable innovations, fBm, and Lévy flights. Their conclusion was that multifractality observed in financial time series was primarily a consequence of the characteristic fat-tailed distribution of the returns. To be specific, Barunik et al. [16] argue that the multifractality of financial time series data partly results from a heavy-tailed α-stable Lévy distribution, suggesting that the observed financial time series data behave more like a fractional Lévy motion than a fractional Brownian motion. Thus, one might ask, what does it mean if one calculates the scaling exponent, theoretically derived from fractional Brownian motion for data that inherently disagrees with this model. Further, the observed multifractality of these datasets might be due to the fact that traditional algorithms, e.g., R/S analysis, cannot deal with α-stable fractional Lévy motions. Thus, we argue that one should aim for determining the scaling exponent of α-stable fractional Lévy motion rather than that of fractional Brownian motion to argue for behavior changes in financial data.

To further clarify this discussion: The term “α-stable” is used to describe a class of probability distributions that has heavy tails and exhibits skewness. It’s the “α” in the “α-stable” that controls the thickness of the tails of the distribution. The lower the “α”, the thicker the tail. When “α = 2”, you have a normal distribution, i.e., fractional Brownian motion, which is the only stable distribution with finite variance. Financial data often exhibits “α-stability” due to the “fat tails” observed in return distributions—that is, extreme events occur more frequently than would be predicted by a normal distribution.

However, it is important to note that while fractional Lévy motion might offer a more accurate representation, it does not fully capture the complexities of financial markets. Real-world financial data are influenced by a multitude of factors, many of which might not be accounted for in the current mathematical models. Nevertheless, in the following, we still aim to show that the stock market data under study is better described by a fractional Lévy motion, and further, that fractional Lévy motion produces different scaling behavior because of its multifractal and/or α-stable aspects. For this reason, we employ the following slightly altered R/S analysis to show this:

The Hurst exponent is a measure for the long-term memory of a time series data and is calculated by R/S Analysis [5]. Following [5,39]:

The *R/S analysis* (Rescaled Range analysis) identifies long-run correlations in time series, yielding one parameter, the *Hurst exponent* “*H*”.

We start with a given signal $\left[x_{1},x_{2},\ldots ,x_{N}\right]$, we then dissect this signal into sliding windows of length *n*, labeled using the index l∈0,1,2,3,…, such that $\left[x_{l+1},x_{l+2},\ldots ,x_{l+n}\right]$.

One finds the average over a period τ (a sub-interval of the sliding window, i.e., 1≤τ≤n), with $k_{l}$ as $l+1\leq k_{l}\leq l+n$ (labeling all possible all elements in each sliding window) and elements *j* in this interval such that $k_{l}\leq j\leq k_{l}+\tau$ and $k_{l}+\tau \leq l+n$ (all possible periods τ starting with an element $k_{l}$):

(11) $${\langle x\rangle }_{\tau ,k_{l}}\;=\;\frac{1}{\tau }\sum_{j=l+k}^{k_{l}+\tau }\;x_{j}\;.$$

Next, an accumulated departure $\delta x\left(i,\tau ,k_{l}\right)$ over a period i∈1,2,…,τ is calculated as:

(12) $$\delta x\left(i,\tau ,k_{l}\right)\;=\;\sum_{j=k_{l}}^{k_{l}+i}\left(x_{j}-{\langle x\rangle }_{\tau ,k_{l}}\right)\;.$$

The range $R_{l}$, which is the difference between maximal and minimal values of the accumulated departure is:

(13) $$\begin{array}{cc} R_{l}\left(\tau ,k_{l}\right)\; & =\;\max_{i}\left[\delta x\left(i,\tau ,k_{l}\right)\right]\;-\;\min_{i}\left[\delta x\left(i,\tau ,k_{l}\right)\right]\;,\\ \; & \mathrm{satisfying}\;\;k_{l}\leq i\leq k_{l}+\tau \;. \end{array}$$

And finally, the standard deviation for each subinterval is:

(14) $$S_{l}\left(\tau ,k_{l}\right)\;=\;\sqrt{\frac{1}{\tau }\sum_{j=k_{l}}^{k_{l}+\tau }{\left[x_{j}-{\langle x\rangle }_{\tau ,k_{l}}\right]}^{2}}\;.$$

The range and the standard deviation are then averaged over all possible (The algorithms that perform R/S analysis find a subset of possible intervals and perform the procedure on this subset.) $k_{l}$ such that:

(15) $$\begin{array}{cc} \; & R_{l}\left(\tau \right)\;=\;\frac{\sum_{k_{l}}R_{l}\left(\tau ,k_{l}\right)}{\mathrm{number}\mathrm{of}\mathrm{different}k_{l}s}\;,\\ \; & \;\mathrm{and}\\ \; & S_{l}\left(\tau \right)\;=\;\frac{\sum_{k_{l}}S_{l}\left(\tau ,k_{l}\right)}{\mathrm{number}\mathrm{of}\mathrm{different}k_{l}s}\;. \end{array}$$

And further, we get the averages over all sliding windows:

(16) $$\begin{array}{cc} \; & \bar{R}\left(\tau \right)\;=\;\frac{\sum_{l}R_{l}\left(\tau \right)}{\mathrm{number}\mathrm{of}\mathrm{different}ls}\;,\\ \; & \;\mathrm{and}\\ \; & \bar{S}\left(\tau \right)\;=\;\frac{\sum_{l}S_{l}\left(\tau \right)}{\mathrm{number}\mathrm{of}\mathrm{different}ls}\;. \end{array}$$

**Figure 3:**
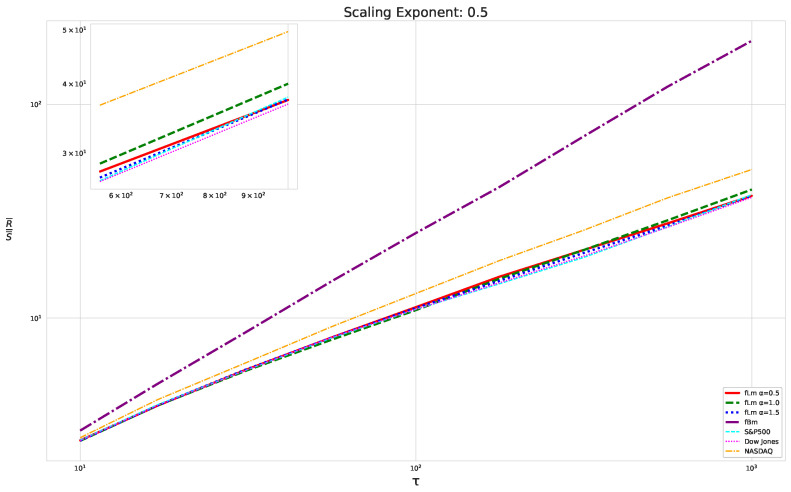
The scaling analysis in the graph offers a comparative view between the three asset datasets: Dow Jones, NASDAQ, and S&P500, as well as fractional Brownian motion. Furthermore, three distinct fractional Lévy motions with a scaling exponent of H=0.5 are also presented. Each fractional Lévy motion depicted has a unique α value (refer to Section 3.1 for more specifics). Note that the R/S ratio displayed is the average R/S ratio, as outlined in Equation (17). To better illustrate the distinctions among the various time series data, we have also provided a zoomed-in view of the final section of the analysis (in terms of the scale τ) in the upper left corner. While this close-up does not include the fractional Brownian motion, it successfully emphasizes the slight differences between the financial time series data, which are otherwise densely clustered.

We then obtain the R/S ratio and the corresponding Hurst exponent as:

(17) $$\frac{\bar{R}\left(\tau \right)}{\bar{S}\left(\tau \right)}\;\propto \;\tau^{H}\;,$$

whereas we only use the this averaged R/S-ratio for the following analysis. Summing up this idea:

We begin our analysis by implementing the Rescaled Range (R/S) analysis over sliding windows across the time series, each containing 1000 consecutive data points. Then, we average the R/S ratio over these sliding windows, as discussed above. We focus on the R/S ratio’s behavior as depicted in the double logarithmic plot in Figure 3 rather than the resulting Hurst exponent. This tactic allows for a comparative study of the scaling behaviors of fractional Lévy motion, fractional Brownian motion, and the financial data under study.

The average range and standard deviation over various sliding windows are modifications that ought to generate a smoother curve. We did this for two reasons:

Firstly, the R/S analysis, applied over numerous τ values on long time series (i.e., classical R/S analysis), is a trusted technique for estimating the scaling exponent. However, comparisons in the log–log space can be challenging due to the R/S ratio’s frequent fluctuations, resulting in occasional deviations even from the theoretical ground truth for known or predefined Hurst exponents associated with fractional Brownian motions.

Secondly, smoother curves allow us to observe the multifractal characteristics of our data, which are often blurred by the fluctuating R/S ratio values. This means we do not see a bent or straight curve when everything fluctuates. Although these multifractal properties and the corresponding fluctuations are inherent to stochastic processes, theory expects a straight and (somehow) smooth line for fractional Brownian motions, indicative of a typical monofractal time series. Further, we expect bent curves for multifractal time series, meaning that the scaling is not the same on all scales but slightly varies from micro to macro scales.

Underpinning our analysis is the Efficient Market Hypothesis (EMH), which posits that financial data, reflecting all available information, is on average unpredictable. This unpredictability infers a Hurst exponent of 0.5—a central value for financial time series behavior under the weak form of EMH [2,40,41]. Following this rationale, the logarithmic price series of security prices should conform to a geometric Brownian motion, transforming into a random walk for the return series [42]. Thus we compare our datasets to fractional Brownian and Lévian motions with scaling exponents of H=0.5.

We illustrate our results on the scaling behavior using the previously discussed modified R/S-approach in Figure 3. The plot displays a linear behavior for fractional Brownian motion, which matches our expectations. Notably, we did not use any smoothing techniques or polynomial fits; we merely connected the points. However, the fractional Lévy motion, despite possessing the same Hurst exponent, exhibits a distinctly different behavior—bent curves indicative of multifractal behavior and a lower scaling exponent than expected. This implies that the curves are not as steep as those of the fractional Brownian motion. Intriguingly, all three financial datasets under analysis align more closely with the fractional Lévy motion, showcasing bent curves and, in the case of the Dow Jones and S&P500, closely matching the curves for fractional Lévy motions (For consistency, our data are restricted to the timeframe from 3 January 1972, to 30 October 2020, which is common to all datasets.).

Therefore, for subsequent analysis, we employ the models from our previous experiments (Section 4) that performed best for fractional Lévy motion. This limits us to the trained CatBoost and MLP models, which performed strongly over all different Lévy motions with window lengths of 200 and 350. CatBoost also topped performance for the fractional Brownian motion within these window lengths.

In Appendix A, we present additional plots illustrating how the scaling behavior and multifractality of fractional Lévy motions change with varying α values.

### 5.2. Results

We analyze the stock market data from three indices in a manner similar to the methodology employed by Alvarez et al. [32], aiming to observe patterns analogous to their study. That is, we use sliding windows over asset data to estimate a time-varying Hurst/scaling exponent.

**Figure 4:**
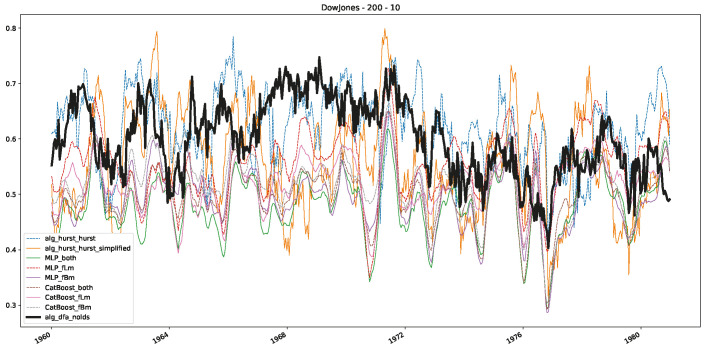
Plot depicting the time-varying DFA and Hurst exponents, as well as the predictions from all trained machine learning models, using a 200-day input window and a 10-day step size between windows, close up for the years 1960–1980.

**Figure 5:**
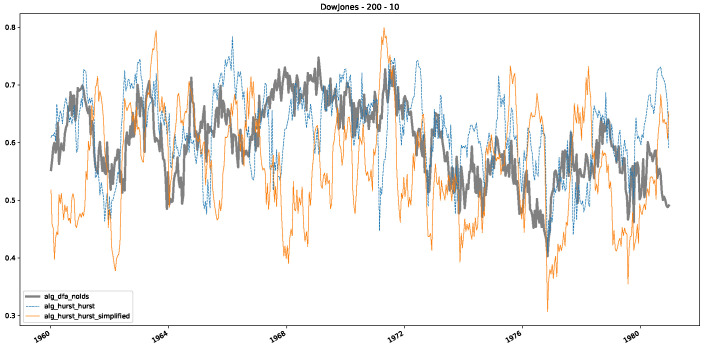
Plot depicting the time-varying DFA and Hurst exponents, using a 200-day input window and a 10-day step size between windows, close up for the years 1960–1980.

**Figure 6:**
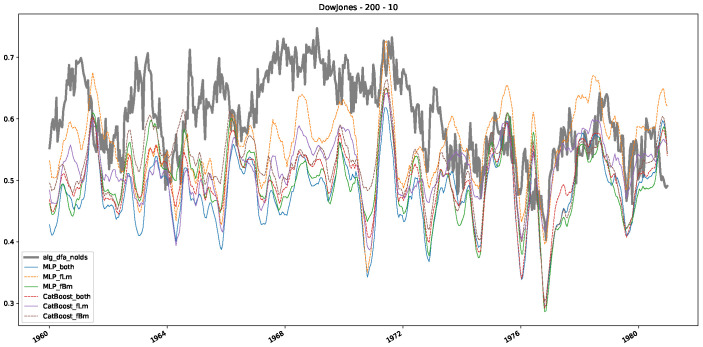
Plot depicting the time-varying DFA and predictions from all trained machine learning models, using a 200-day input window and a 10-day step size between windows, close up for the years 1960–1980.

In addition to their chosen assets, we incorporate NASDAQ data into our analysis. As indicated in the previous Section 5.1, we focus on the models that most accurately portray the scaling behavior of fractional Lévy motions, namely the trained MLP and CatBoost models. Coincidentally, CatBoost also excels in identifying the scaling exponent of fractional Brownian motions. Our findings are illustrated in Figure 4, Figure 5 and Figure 6, which bear resemblance to Figures 6 and 7 from [32], indicating a similar (or the same) shift in the DFA “scaling exponent” around 1972. However, the other methods used to detect this shift or similar behavior did not reproduce the shift observed in [32], which we also observe for all the other configurations (sliding window sizes and in-between steps) in Appendix B. Considering our earlier experiments and validation from Section 5.1 and from the literature [16], suggesting that the stock market follows a fractional Lévy motion rather than a fractional Brownian motion, we conclude that DFA may not accurately represent the scaling exponent of stock market data. The interested reader is referred to Appendix B, where we show the plots for the full range of our experiments, all available years, and all three assets. In the current section, we only used an excerpt from the Dow Jones from 1960 to 1980 to emphasize trends and correlations of the time-varying scaling exponents.

**Figure 7:**
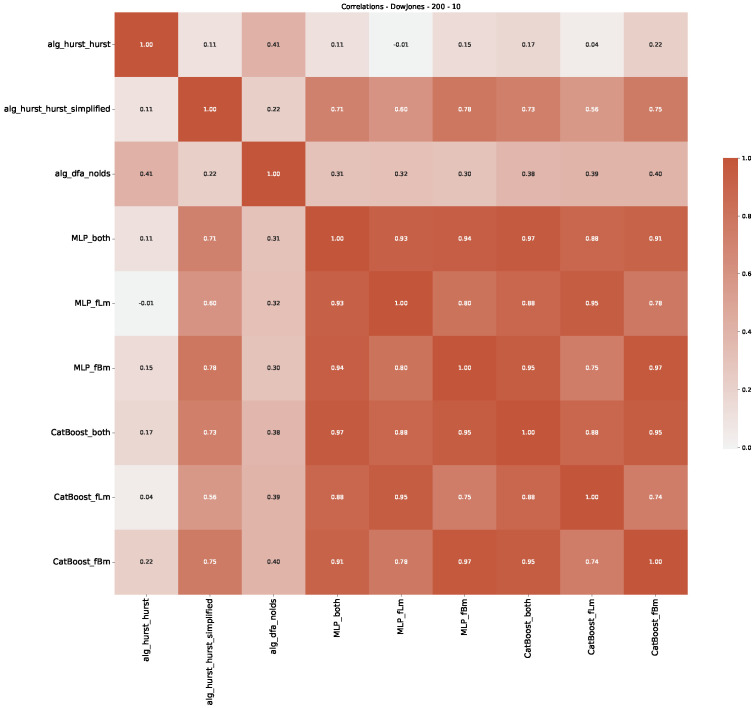
Correlation plot showing the relationships between the DFA, various Hurst exponent estimation methods, and the predictions of all trained machine learning models for the Dow Jones index, using a 200-day rolling window size and a step size of 10 days, close up for the years 1960–1980.

Furthermore, we examine the correlations between DFA and the trained machine learning models used to estimate the scaling exponent of time series data for the Dow Jones Index in Figure 7 (This correlation matrix takes into account all available years for the Dow Jones index, not only the interval 1960 to 1980). Notably, the machine learning models show strong intercorrelations, while their correlation with the employed “classical” algorithms is weak. Meaning that the trained machine-learning models display similar patterns as shown in Figure 6. What is interesting here is that the simplified R/S algorithm from the python package hurst (denoted as alg_hurst_hurst_simplified) is closer to the results from the trained machine learning models than the other classical algorithms. We see this in both the plot depicting all sliding window scaling exponents of all algorithms and machine learning models (Figure 4) and the correlation matrix (Figure 7). This simplified R/S analysis correlates stronger with the machine learning algorithms’ estimates than the other classical algorithms. We also observe this or very similar behavior for all the other configurations and assets in Appendix B.

Moreover, we conduct an analogous analysis on the S&P500 and NASDAQ, including results for various input window lengths and step sizes (350-day window and 10, 50-day step sizes) in Appendix B. The results for these assets align with our findings for the Dow Jones, showing that DFA does not correlate well with the trained machine learning models and that these models offer a different perspective on the estimated scaling exponents. It is worth mentioning that we observed an increased correlation between machine learning algorithms and DFA in estimating a scaling parameter for NASDAQ data. This concurs with our earlier discussion from Section 5.1 and Figure 3, where the discussed NASDAQ data are closer to that of a fractional Brownian motion than the other datasets. Therefore, this increased correlation for NASDAQ suggests that its data are indeed more akin to a fractional Brownian motion, leading to greater alignment between DFA and the machine learning models’ estimates, given that classical methods perform better for data resembling fractional Brownian motion.

### 5.3. Summary & Discussion

In this article, we discuss how machine learning models can be used to estimate the scaling exponents of time series data. We showed and validated our ideas with two experiments, the first one was to show how well-trained machine learning models can estimate the scaling exponent of stochastic processes, in our case for fractional Brownian and fractional Lévy motions, compared to classical algorithms to estimate the scaling exponent of a time series data. The second one uses well-performing machine learning models to estimate the Hurst/scaling exponents of financial time series data and compares the results to well-known algorithms and results from the literature.

Our first experiment on estimating the scaling exponent of stochastic processes shows that classical algorithms are outperformed by the trained machine learning models, especially by sophisticated boosting algorithms such as LightGBM or CatBoost or just a plain neural network approach in the form of a Multi-Layer Perceptron. Reflecting on this experiment’s results, i.e., showing consistent evidence that machine learning algorithms can estimate the scaling exponent of time series with more accuracy than classical algorithms in most cases, we conclude that the latter may not provide reliable scaling exponents for stock market data. This conclusion is based on the result, that classical algorithms do not particularly perform well for the case of fractional Lévy motions, and further evidence, that the stock market under study follows rather a fractional Lévy motion than a fractional Brownian motion.

Admittedly, these classical algorithms to estimate the scaling exponent of time series data, and modified versions of them [32,43,44,45,46,47], have long been employed to analyze stock markets, and undoubtedly, they have offered valuable insights over time. However, with the advent and rise of artificial intelligence, finance professionals may benefit from augmenting their scaling exponent estimates by incorporating machine learning models into their analytical repertoire alongside traditional methods.

We further need to discuss results from the literature to estimate a Hurst/scaling exponent using machine learning approaches. We observe that results from the past do not explicitly state how they generated their training data or performed the training [9]. Further, to the best of our knowledge, there is no study incorporating the scaling exponent of other stochastic processes than fractional Brownian motions, and/or obtained from real life data via a classical algorithm. Moreover, many articles are not using a regression but a classification approach, thus these approaches cannnot estimate a continuous scaling exponent [9,21], and oftentimes the estimation is restricted to scaling exponents of only 0.5 and above, thus leaving out the part of heavily fluctuating time series data. Thus we consider our approach and the corresponding code, the trained models and all training datasets, a big contribution to the research on stochastic processes and related real life data [31].

The simplified R/S analysis seems to more accurately reflect the scaling behavior learned by the machine learning algorithms, as demonstrated by the correlation plots in Section 5.2 and Appendix B. Coinciding with this result: The simplified algorithm outperforms other traditional algorithms in identifying the scaling exponent for fractional Lévy motion with a Lévy index of α=1.0. Further, in the experiment discussed in Section 4.3.3, the simplified Hurst exponent also slightly surpasses the performance of the best machine learning algorithms for window lengths of 200 and 350 (We should note that for this comparison, we disregarded the variability of the error and compared only the average errors. When considering the corresponding variability, we find that the best machine learning algorithms and the simplified version of R/S analysis perform very similarly.).

In the case of fractional Brownian motion, the simplified R/S analysis ranks well among the traditional algorithms. While it does not perform as well as DFA for lengths of 100, 200, and 350, it is the second best. Furthermore, the increased correlation with the machine learning algorithms used for the analysis of financial data in Section 5.2 leads us to conclude that this simplified version of R/S analysis is best suited among the classical algorithms for analyzing stock market data in a sliding window manner.

And finally, we need to mention an odd discovery: The models that were trained exclusively on fractional Lévy motions did not perform optimally when applied to fractional Lévy motions with tested Lévy indices α>0.5 (Section 4.3.3 and Section 4.3.4). This may be attributable to the increased frequency of extreme events within these datasets, and the fact that these datasets are closer to fractional Brownian motions than, e.g., the fractional Lévy motion with an α=0.5. As a result, models trained on fractional Brownian motion might provide better estimates of the scaling exponent since their training data are not obscured by these extreme events.

## 6. Conclusions

Our article presents a machine learning approach to identify the Hurst or scaling exponent of time series data. We employed both artificial datasets and real-life datasets to demonstrate the applicability of our ideas. The following steps were performed to train and validate our models and ideas:

1. We trained a range of machine learning models on both fractional Brownian and fractional Lévy motions with different Hurst/scaling exponents and different Lévy indices. We used the known scaling exponent as the ground truth for the value to be predicted by the machine learning algorithms, i.e., the output of the models. The features, or the input, are time series data from the discussed stochastic processes scaled to the unit interval $\left[0;1\right]$.
2. We validated the trained models for different lengths of input windows using, again, fractional Brownian and fractional Lévy motions. The results show that in most cases the trained machine learning models outperform classical algorithms (such as R/S analysis) to estimate the scaling exponent of both fractional Brownian and fractional Lévy motions.
3. We then took three asset time series, i.e., Dow Jones, S&P500, and NASDAQ, and applied a slightly modified version of R/S analysis to these datasets to show that these data signals are more akin to fractional Lévy motions than fractional Brownian motions in nature. The reason for doing this was to argue that certain classical algorithms cannot correctly estimate the scaling exponents of these datasets because, as shown in the previous step, compared to the trained models, they suffer from large errors in estimating the scaling exponent of fractional Lévy motions.
4. In a final step, we analyzed the scaling exponent of the previously named three assets in a sliding window manner, to show and discuss the applicability of the trained models and classical algorithms to estimate the scaling behavior of time series data. Our research shows that results from the literature might be wrong in estimating the scaling exponent using detrended fluctuation analysis (DFA) and drawing conclusions from it. To do this, we first reconstructed the scaling behavior using DFA, which coincides with the results from the literature. We then found that the trained machine learning algorithms do not reproduce the scaling behavior from the literature, even though we showed that the assets under study are closer to a fractional Lévy motion, and that our trained models can better estimate the scaling exponent of stochastic processes like these.

However, since our results show that classical methods to estimate the scaling behavior of fractional Lévy motions and financial data might be inherently flawed, we recommend using the developed ideas and trained models (All our trained models are available in a corresponding GitHub repository using Python.). In the authors’ opinion, finance analysts will not stop using classical tools and algorithms to estimate the scaling behavior of assets and their predictability in the foreseeable future. Thus, we want to emphasize that using our trained models might provide a benefit for doing so. Furthermore, given a larger set of assets and different experimental designs, one should further test our ideas for their validity. However, the authors are confident that the presented ideas will continue to outperform classical algorithms since classical algorithms are almost always based on the concept of fractional Brownian motions, and real-life time series data are hardly ever a perfect case of a theoretical concept.

Our trained machine learning algorithms apply to any process where one can calculate a scaling exponent from time series data and thus can be used as a substitute for calculating the Hurst exponent in environmental applicationss [48,49] or engineering [50].

Finally, we want to state that in the authors’ opinion, the presented machine learning approach might be improved by employing a sophisticated recurrent neural network architecture based on LSTM [51] or GRU [52] neural network cells. Further, as done in an earlier work of the corresponding author, one might test how the trained machine learning models used to estimate the scaling behavior of time series might effectively ascertain the predictability of time series data at different points in time [53].

## Figures and Tables

**Table 1:** The CV-$r^{2}$ scores for all trained machine learning models and various types of training data are presented, with the best results for each type of training data emphasized in bold font. It should be noted that we have not included the errors for both Ridge and Lasso regression, as these regressors yield very low scores across all datasets. Furthermore, as will be demonstrated later, these regressors are unable to produce meaningful values for the Hurst exponent.

Window Length and Type of Training Data	CV-Score Ridge Regressor	CV-Score Lasso Regressor	CV-Score AdaBoost Regressor	CV-Score CatBoost Regressor	CV-Score LightGBM Regressor	CV-Score MLP Regressor
**10, fBm**	<0.0001	<0.0001	0.29876	0.47271	**0.47396**	0.45155
**25, fBm**	<0.0001	<0.0001	0.53221	**0.73295**	0.72234	0.69416
**50, fBm**	<0.0001	<0.0001	0.66082	**0.84480**	0.84369	0.82256
**100, fBm**	<0.0001	<0.0001	0.73564	**0.91811**	0.91329	0.90260
**10, fLm**	<0.0001	<0.0001	0.29913	0.41482	**0.42049**	0.41858
**25, fLm**	<0.0001	<0.0001	0.36256	**0.53270**	0.52886	0.52027
**50, fLm**	<0.0001	<0.0001	0.40205	**0.60694**	0.59713	0.56785
**100, fLm**	<0.0001	<0.0001	0.42368	**0.65468**	0.64698	0.60597
**10, both**	<0.0001	<0.0001	0.29751	0.41519	**0.41749**	0.41090
**25, both**	<0.0001	<0.0001	0.43414	**0.59296**	0.58660	0.55943
**50, both**	<0.0001	<0.0001	0.51247	**0.69218**	0.68393	0.64834
**100, both**	<0.0001	<0.0001	0.56500	**0.76081**	0.75103	0.73408

**Figure A1:**
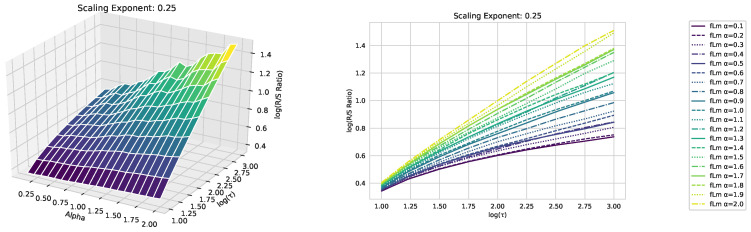
Plots depicting the different scaling behaviors of fractional Lévy motion with varying α, for a fixed scaling exponent of H=0.25.

**Figure A2:**
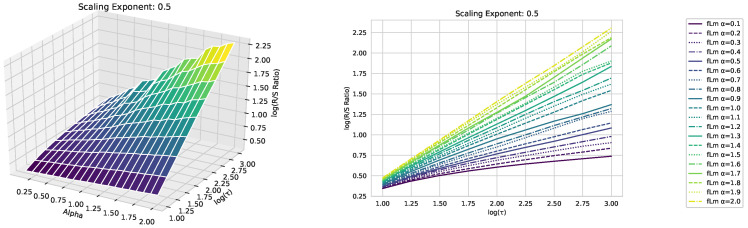
Plots depicting the different scaling behaviors of fractional Lévy motion with varying α, for a fixed scaling exponent of H=0.5.

**Figure A3:**
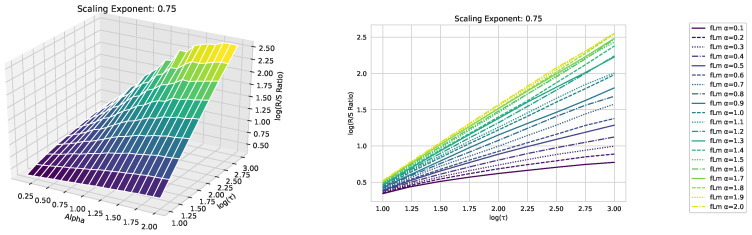
Plots depicting the different scaling behaviors of fractional Lévy motion with varying α, for a fixed scaling exponent of H=0.75.

**Figure A4:**
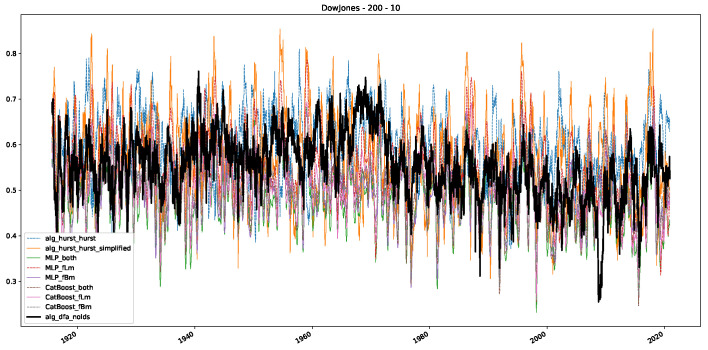
Plot depicting the time-varying DFA and Hurst exponents, as well as the predictions from trained machine learning models, using a 200-day input window and a 10-day step size between windows for the Dow Jones daily close values.

**Figure A5:**
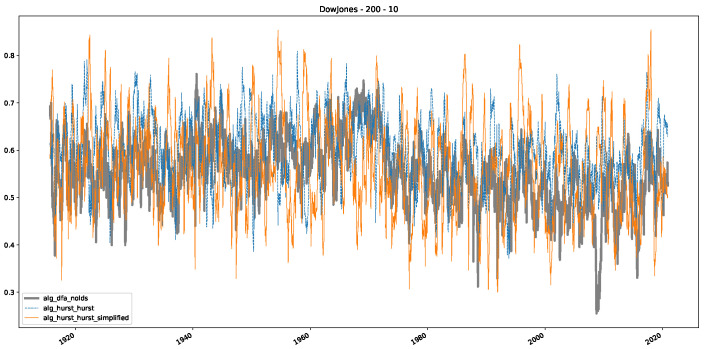
Plot depicting the time-varying DFA and Hurst exponents, using a 200-day input window and a 10-day step size between windows for the Dow Jones daily close values.

**Figure A6:**
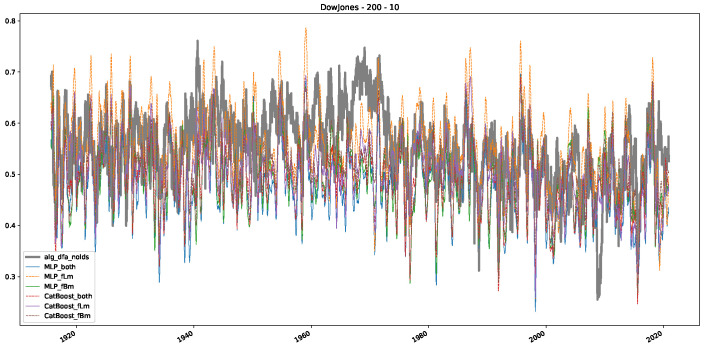
Plot depicting the time-varying DFA and predictions from all trained machine learning models, using a 200-day input window and a 10-day step size between windows for the Dow Jones daily close values.

**Figure A7:**
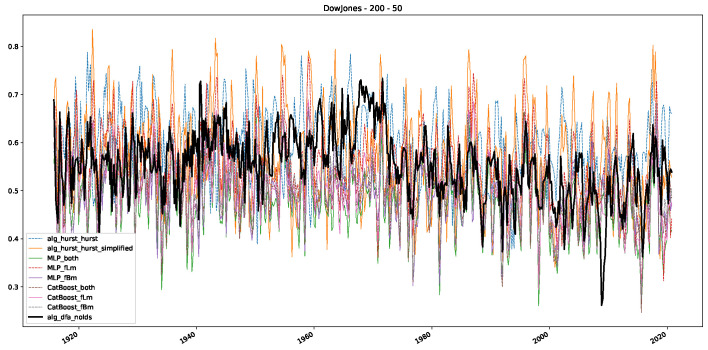
Plot depicting the time-varying DFA and Hurst exponents, as well as the predictions from all trained machine learning models, using a 200-day input window and a 50-day step size between windows for the Dow Jones daily close values.

**Figure A8:**
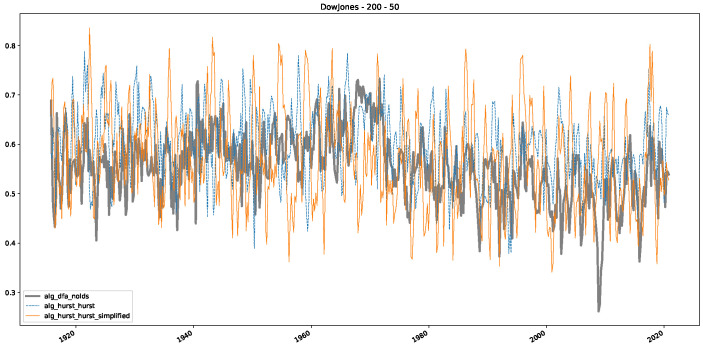
Plot depicting the time-varying DFA and Hurst exponents, using a 200-day input window and a 50-day step size between windows for the Dow Jones daily close values.

**Figure A9:**
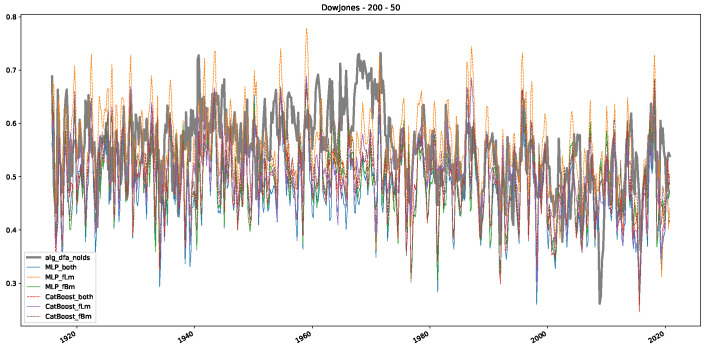
Plot depicting the time-varying DFA and predictions from all trained machine learning models, using a 200-day input window and a 50-day step size between windows for the Dow Jones daily close values.

**Figure A10:**
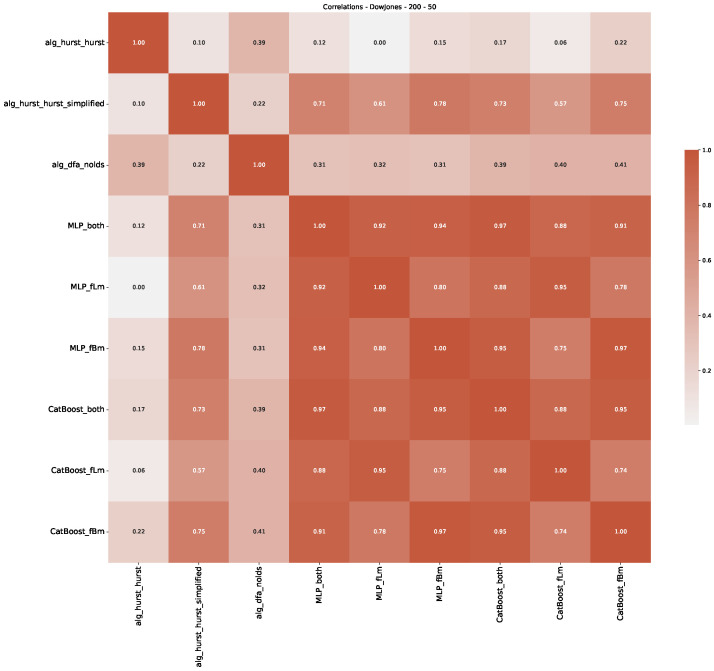
Correlation plot showing the relationships between the DFA, various Hurst exponent estimation methods, and the predictions of all trained machine learning models for the Dow Jones index, using a 200-day rolling window size and a step size of 50 days for the Dow Jones daily close values.

**Figure A11:**
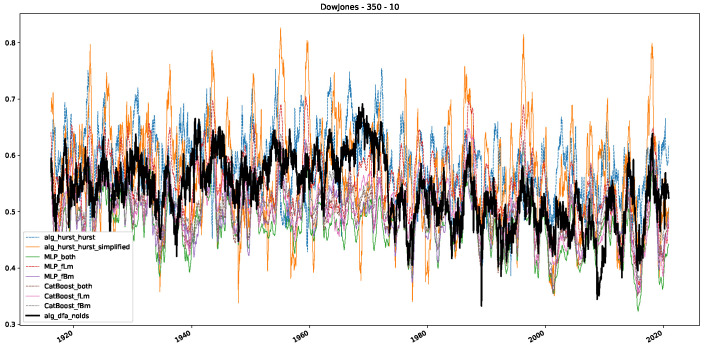
Plot depicting the time-varying DFA and Hurst exponents, as well as the predictions from all trained machine learning models, using a 350-day input window and a 10-day step size between windows for the Dow Jones daily close values.

**Figure A12:**
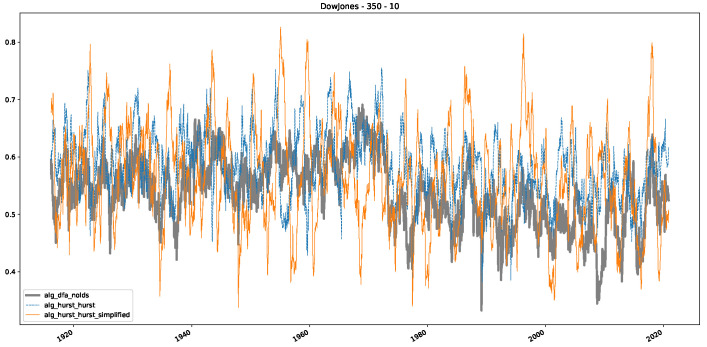
Plot depicting the time-varying DFA and Hurst exponents, using a 350-day input window and a 10-day step size between windows for the Dow Jones daily close values.

**Figure A13:**
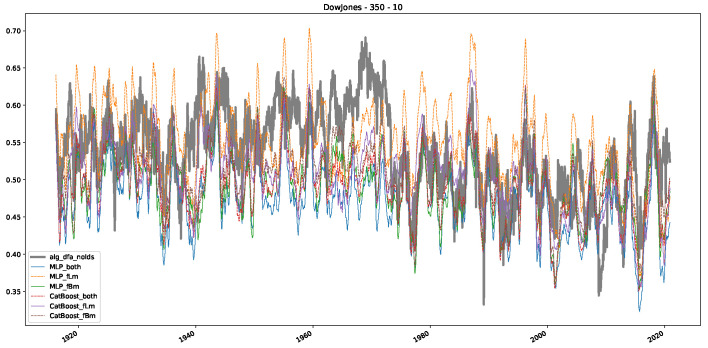
Plot depicting the time-varying DFA and predictions from all trained machine learning models, using a 350-day input window and a 10-day step size between windows for the Dow Jones daily close values.

**Figure A14:**
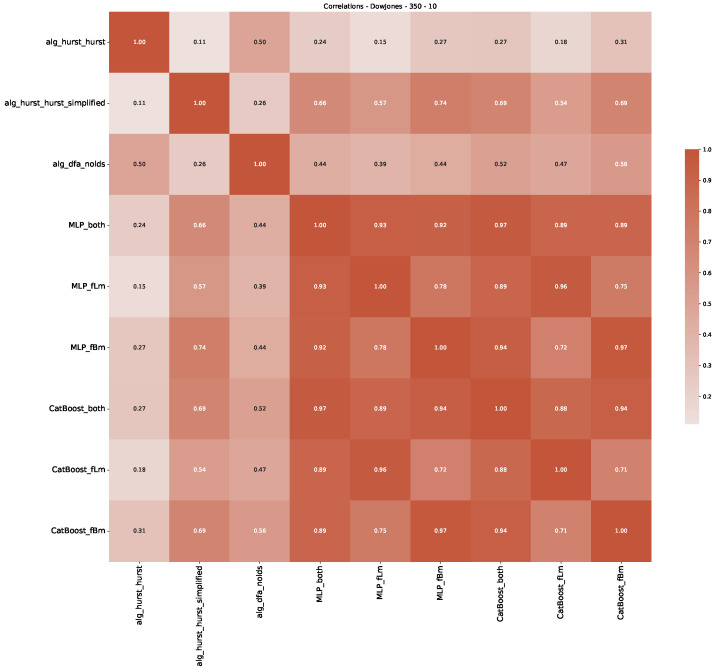
Correlation plot showing the relationships between the DFA, various Hurst exponent estimation methods, and the predictions of all trained machine learning models for the Dow Jones index, using a 350-day rolling window size and a step size of 10 days for the Dow Jones daily close values.

**Figure A15:**
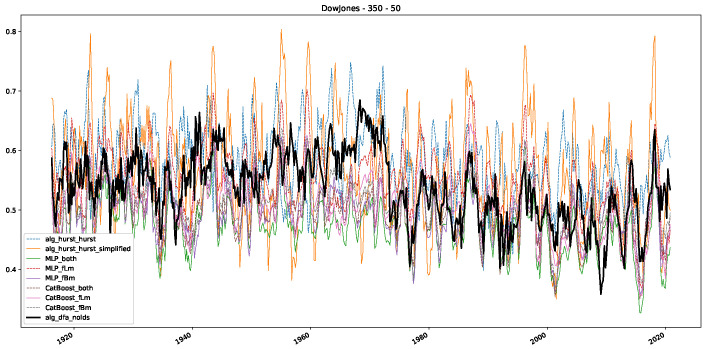
Plot depicting the time-varying DFA and Hurst exponents, as well as the predictions from all trained machine learning models, using a 350-day input window and a 50-day step size between windows for the Dow Jones daily close values.

**Figure A16:**
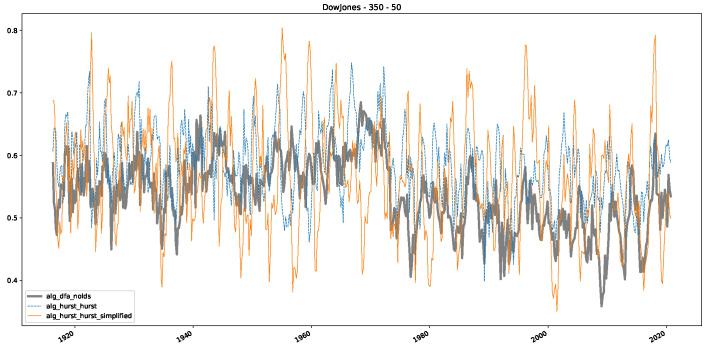
Plot depicting the time-varying DFA and Hurst exponents, using a 350-day input window and a 50-day step size between windows for the Dow Jones daily close values.

**Figure A17:**
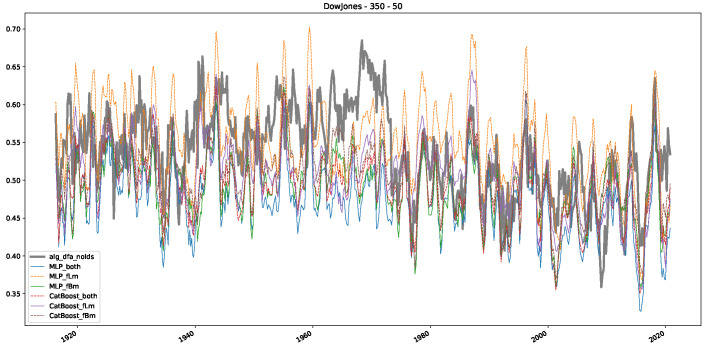
Plot depicting the time-varying DFA and predictions from all trained machine learning models, using a 350-day input window and a 50-day step size between windows for the Dow Jones daily close values.

**Figure A18:**
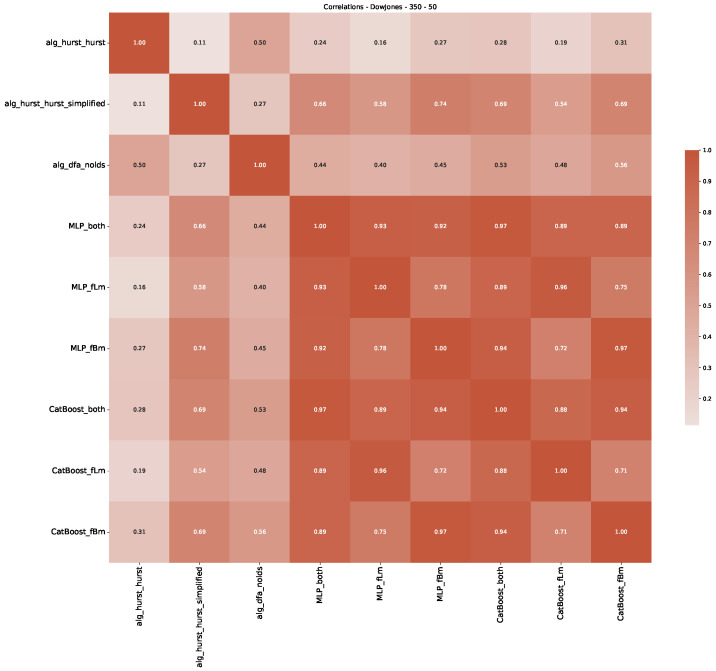
Correlation plot showing the relationships between the DFA, various Hurst exponent estimation methods, and the predictions of all trained machine learning models for the Dow Jones index, using a 350-day rolling window size and a step size of 50 days for the Dow Jones daily close values.

**Figure A19:**
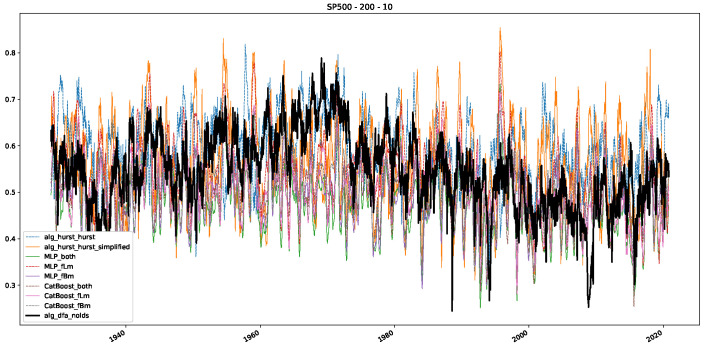
Plot depicting the time-varying DFA and Hurst exponents, as well as the predictions from all trained machine learning models, using a 200-day input window and a 10-day step size between windows for the S&P500 daily close values.

**Figure A20:**
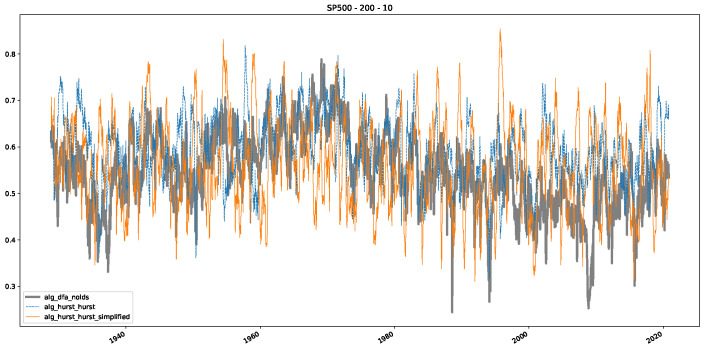
Plot depicting the time-varying DFA and Hurst exponents, using a 200-day input window and a 10-day step size between windows for the S&P500 daily close values.

**Figure A21:**
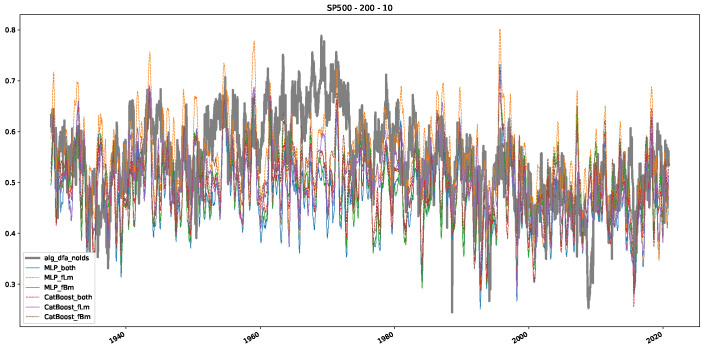
Plot depicting the time-varying DFA and predictions from all trained machine learning models, using a 200-day input window and a 10-day step size between windows for the S&P500 daily close values.

**Figure A22:**
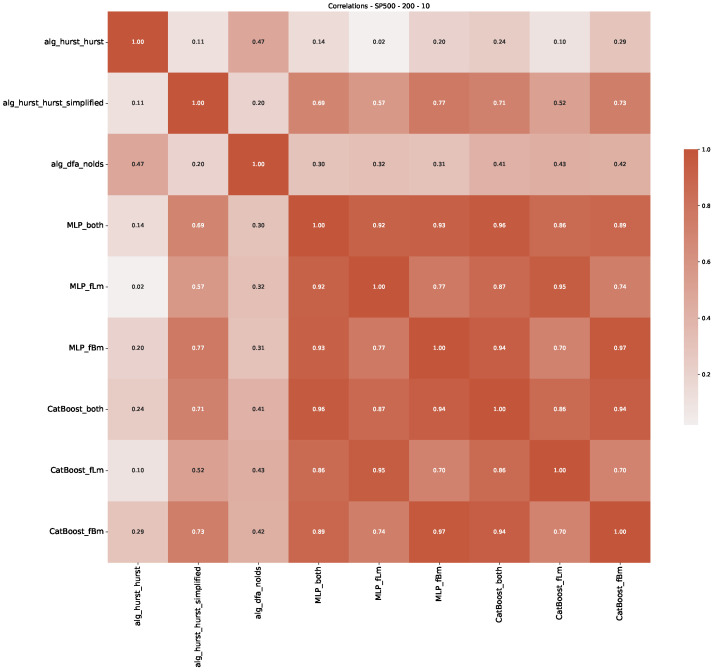
Correlation plot showing the relationships between the DFA, various Hurst exponent estimation methods, and the predictions of all trained machine learning models for the Dow Jones index, using a 200-day rolling window size and a step size of 10 days for the S&P500 daily close values.

**Figure A23:**
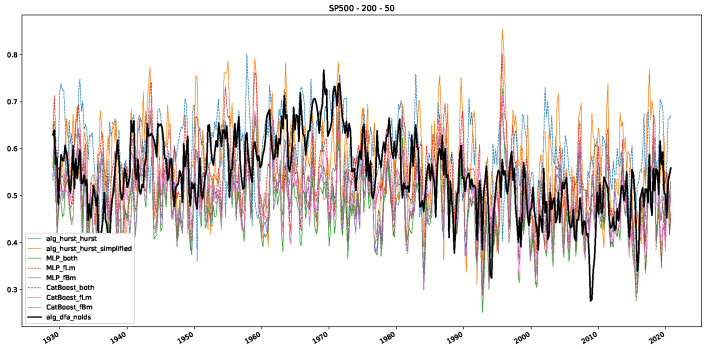
Plot depicting the time-varying DFA and Hurst exponents, as well as the predictions from all trained machine learning models, using a 200-day input window and a 50-day step size between windows for the S&P500 daily close values.

**Figure A24:**
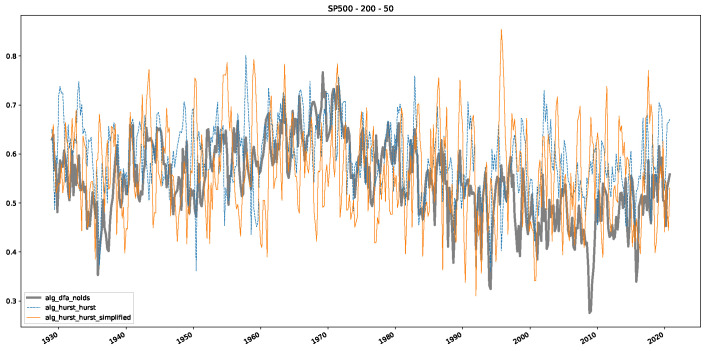
Plot depicting the time-varying DFA and Hurst exponents, using a 200-day input window and a 50-day step size between windows for the S&P500 daily close values.

**Figure A25:**
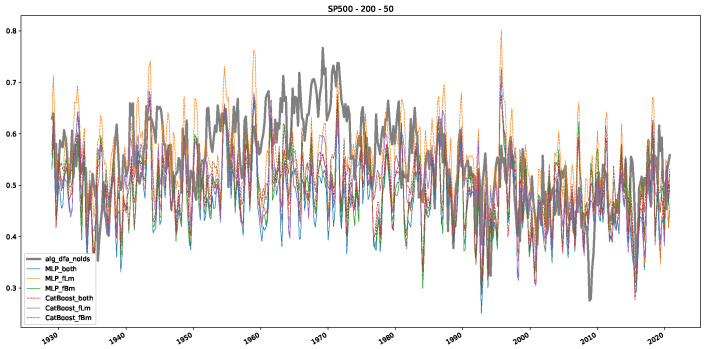
Plot depicting the time-varying DFA and predictions from all trained machine learning models, using a 200-day input window and a 50-day step size between windows for the S&P500 daily close values.

**Figure A26:**
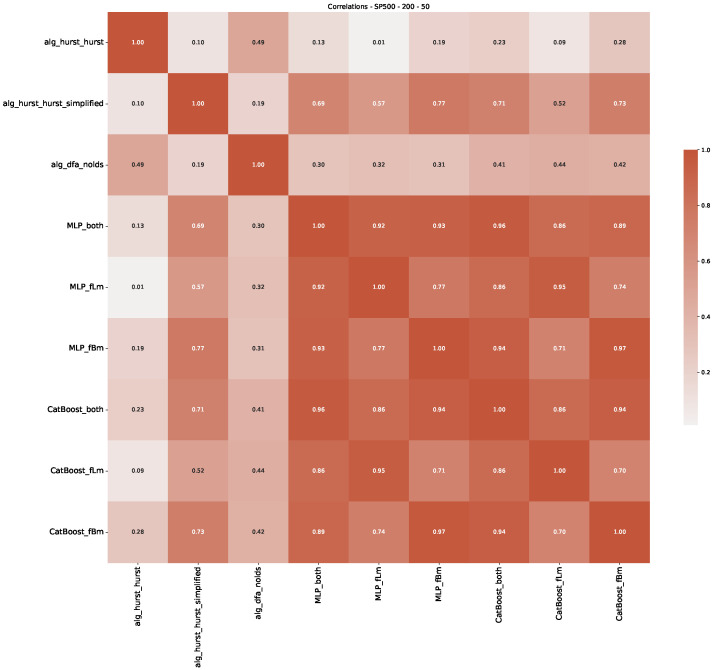
Correlation plot showing the relationships between the DFA, various Hurst exponent estimation methods, and the predictions of all trained machine learning models for the Dow Jones index, using a 200-day rolling window size and a step size of 50 days for the S&P500 daily close values.

**Figure A27:**
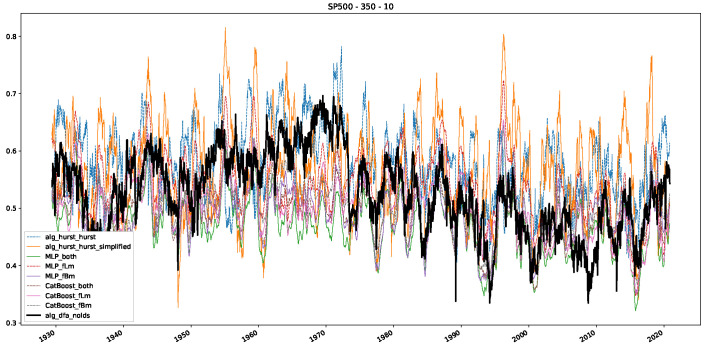
Plot depicting the time-varying DFA and Hurst exponents, as well as the predictions from all trained machine learning models, using a 350-day input window and a 10-day step size between windows for the S&P500 daily close values.

**Figure A28:**
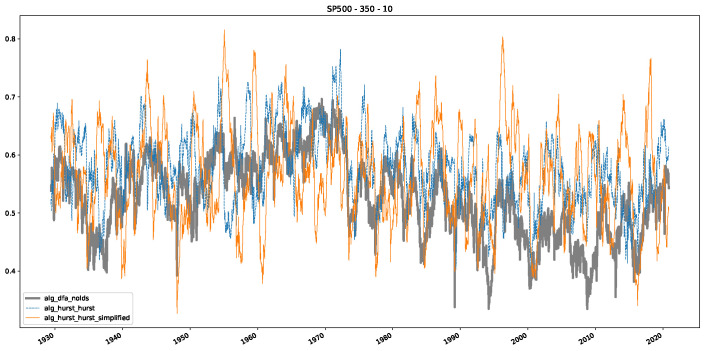
Plot depicting the time-varying DFA and Hurst exponents, using a 350-day input window and a 10-day step size between windows for the S&P500 daily close values.

**Figure A29:**
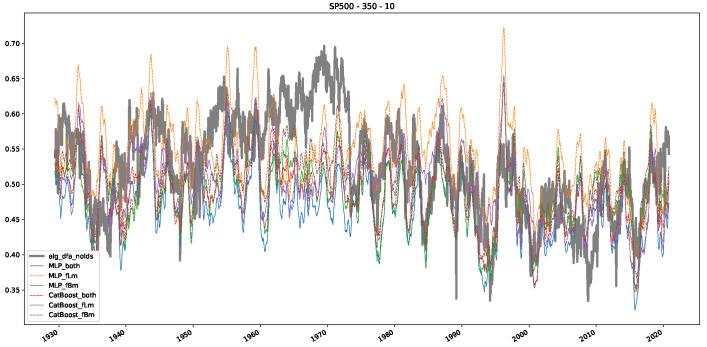
Plot depicting the time-varying DFA and predictions from all trained machine learning models, using a 350-day input window and a 10-day step size between windows for the S&P500 daily close values.

**Figure A30:**
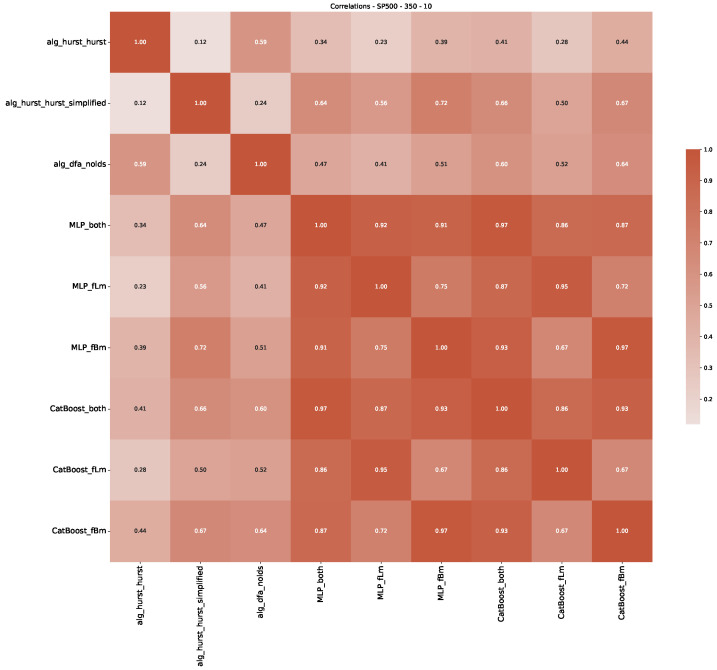
Correlation plot showing the relationships between the DFA, various Hurst exponent estimation methods, and the predictions of all trained machine learning models for the Dow Jones index, using a 350-day rolling window size and a step size of 10 days for the S&P500 daily close values.

**Figure A31:**
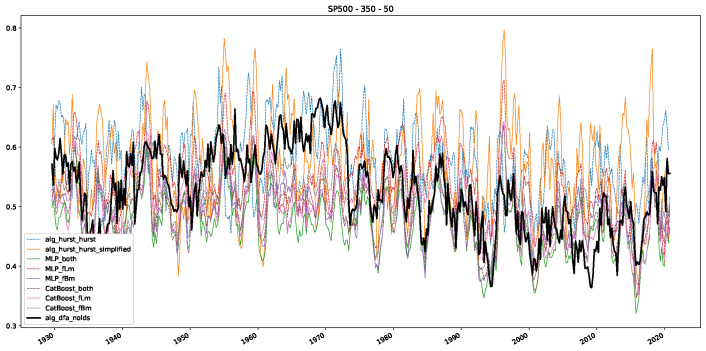
Plot depicting the time-varying DFA and Hurst exponents, as well as the predictions from all trained machine learning models, using a 350-day input window and a 50-day step size between windows for the S&P500 daily close values.

**Figure A32:**
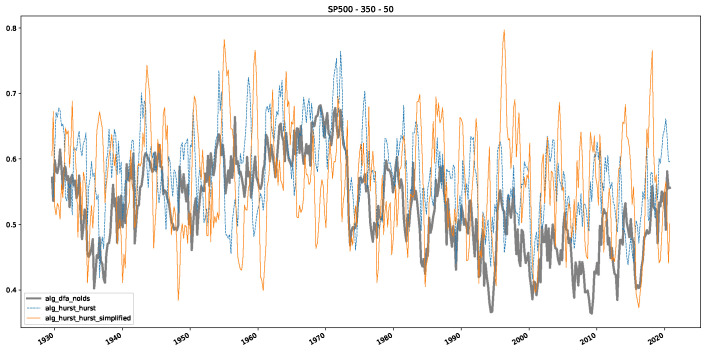
Plot depicting the time-varying DFA and Hurst exponents, using a 350-day input window and a 50-day step size between windows for the S&P500 daily close values.

**Figure A33:**
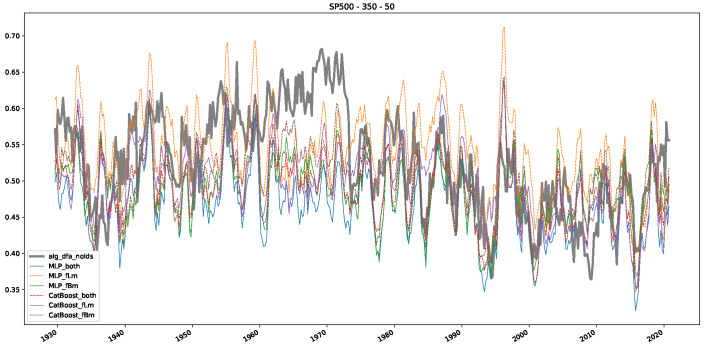
Plot depicting the time-varying DFA and predictions from all trained machine learning models, using a 350-day input window and a 50-day step size between windows for the S&P500 daily close values.

**Figure A34:**
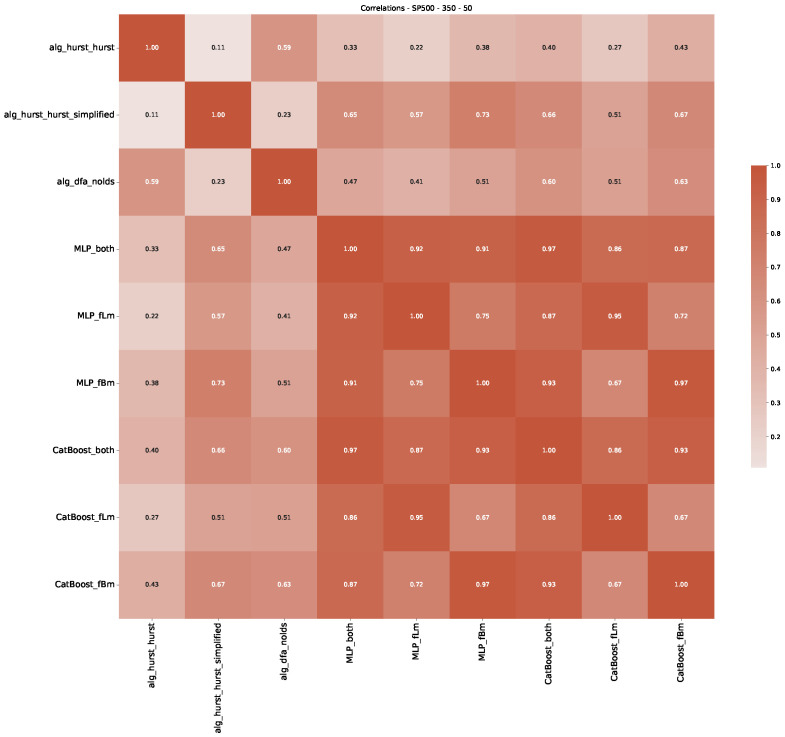
Correlation plot showing the relationships between the DFA, various Hurst exponent estimation methods, and the predictions of all trained machine learning models for the Dow Jones index, using a 350-day rolling window size and a step size of 50 days for the S&P500 daily close values.

**Figure A35:**
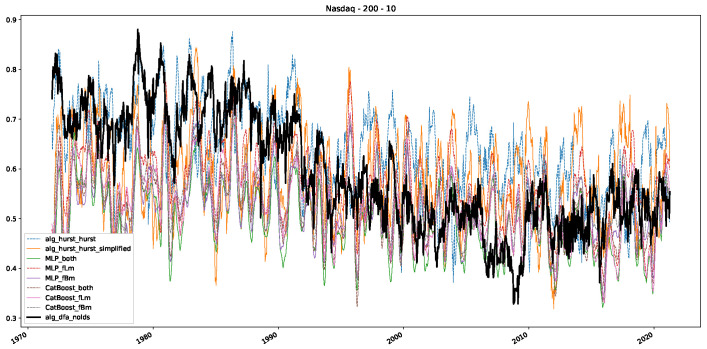
Plot depicting the time-varying DFA and Hurst exponents, as well as the predictions from all trained machine learning models, using a 200-day input window and a 10-day step size between windows for the NASDAQ daily close values.

**Figure A36:**
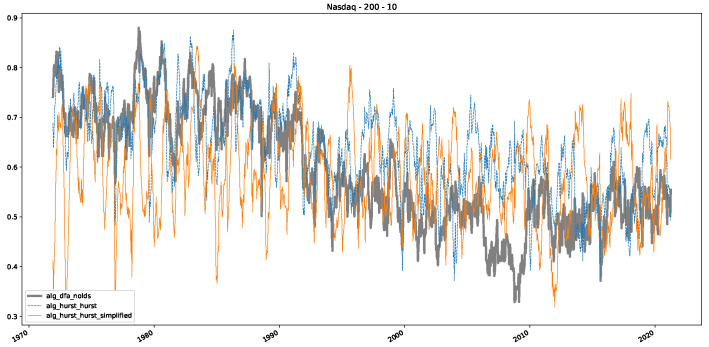
Plot depicting the time-varying DFA and Hurst exponents, using a 200-day input window and a 10-day step size between windows for the NASDAQ daily close values.

**Figure A37:**
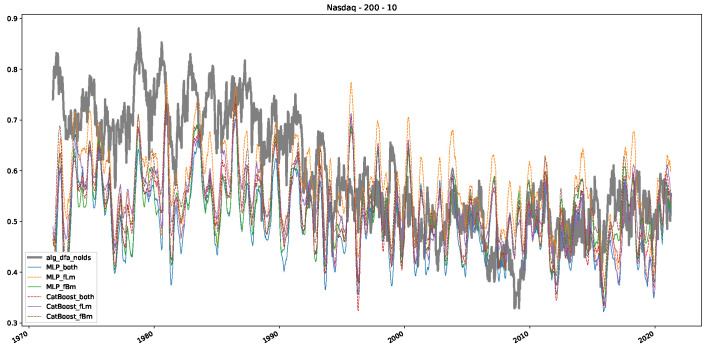
Plot depicting the time-varying DFA and predictions from all trained machine learning models, using a 200-day input window and a 10-day step size between windows for the NASDAQ daily close values.

**Figure A38:**
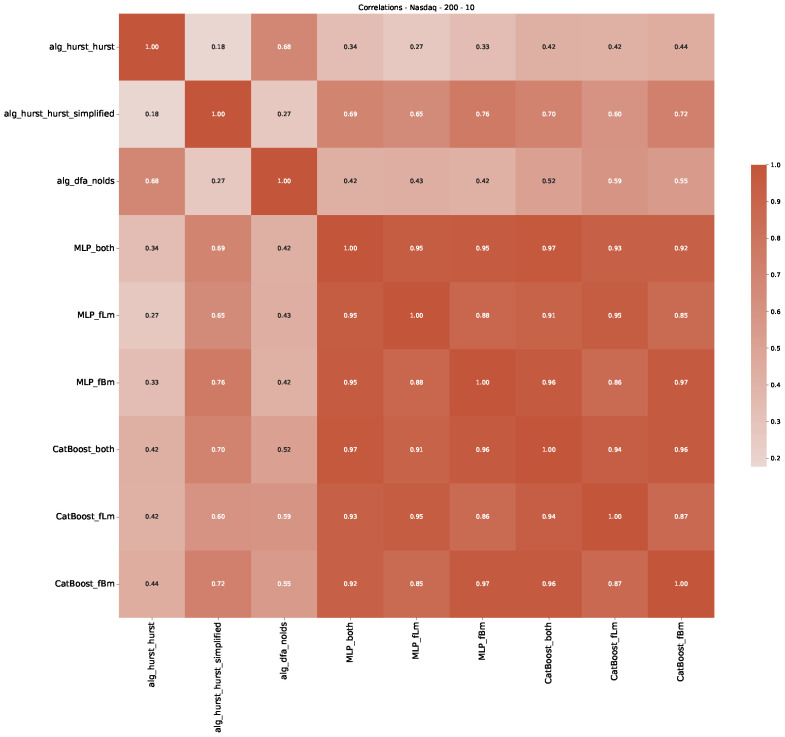
Correlation plot showing the relationships between the DFA, various Hurst exponent estimation methods, and the predictions of all trained machine learning models for the Dow Jones index, using a 200-day rolling window size and a step size of 10 days for the NASDAQ daily close values.

**Figure A39:**
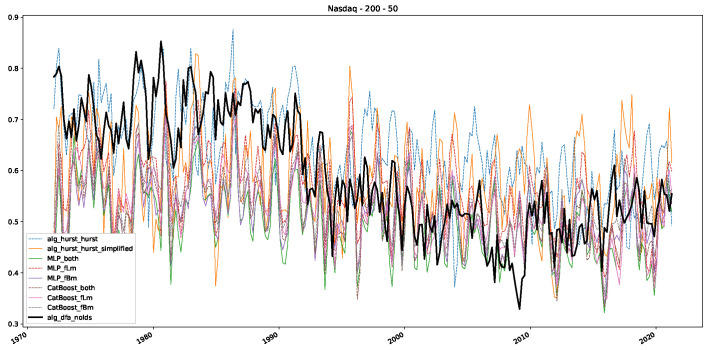
Plot depicting the time-varying DFA and Hurst exponents, as well as the predictions from all trained machine learning models, using a 200-day input window and a 50-day step size between windows for the NASDAQ daily close values.

**Figure A40:**
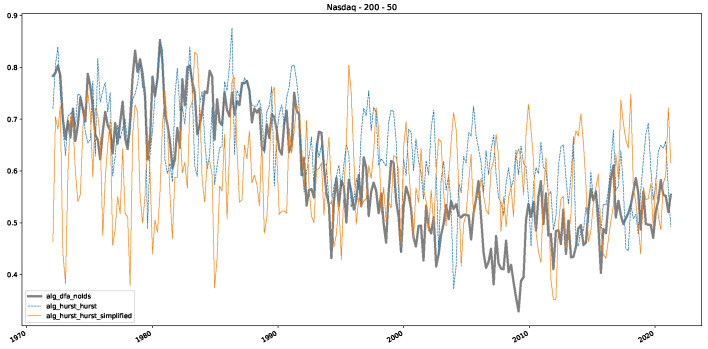
Plot depicting the time-varying DFA and Hurst exponents, using a 200-day input window and a 50-day step size between windows for the NASDAQ daily close values.

**Figure A41:**
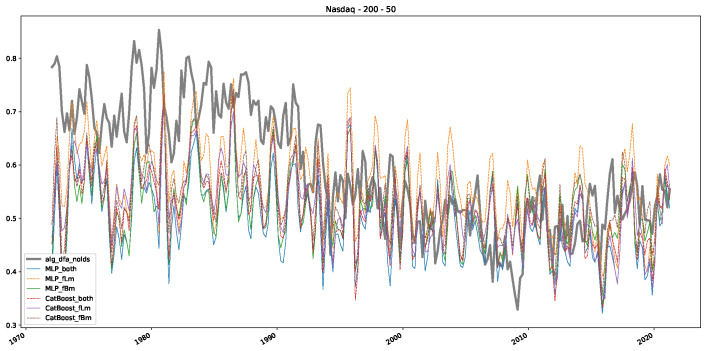
Plot depicting the time-varying DFA and predictions from all trained machine learning models, using a 200-day input window and a 50-day step size between windows for the NASDAQ daily close values.

**Figure A42:**
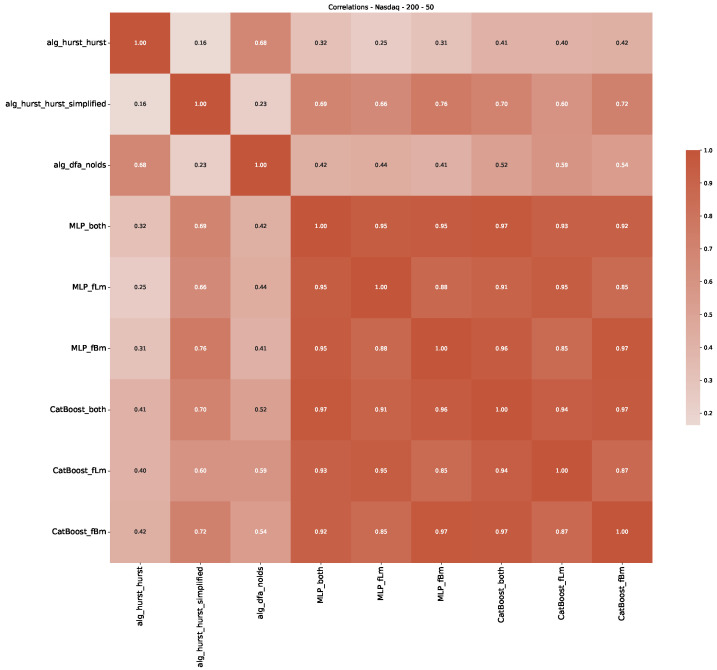
Correlation plot showing the relationships between the DFA, various Hurst exponent estimation methods, and the predictions of all trained machine learning models for the Dow Jones index, using a 200-day rolling window size and a step size of 50 days for the NASDAQ daily close values.

**Figure A43:**
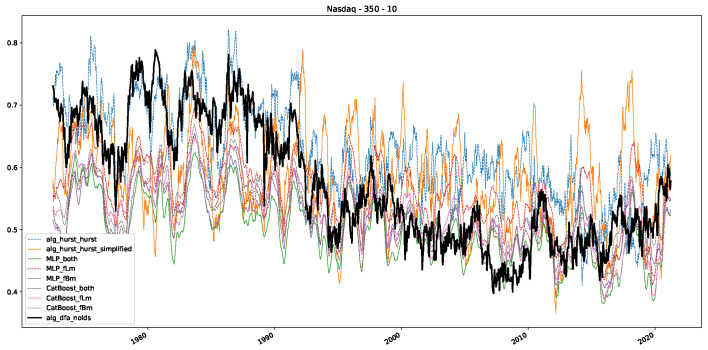
Plot depicting the time-varying DFA and Hurst exponents, as well as the predictions from all trained machine learning models, using a 350-day input window and a 10-day step size between windows for the NASDAQ daily close values.

**Figure A44:**
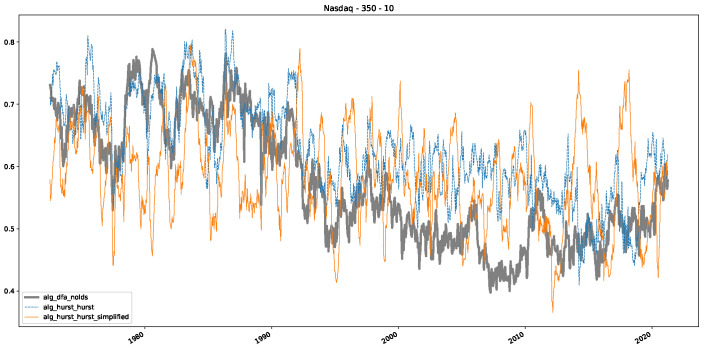
Plot depicting the time-varying DFA and Hurst exponents, using a 350-day input window and a 10-day step size between windows for the NASDAQ daily close values.

**Figure A45:**
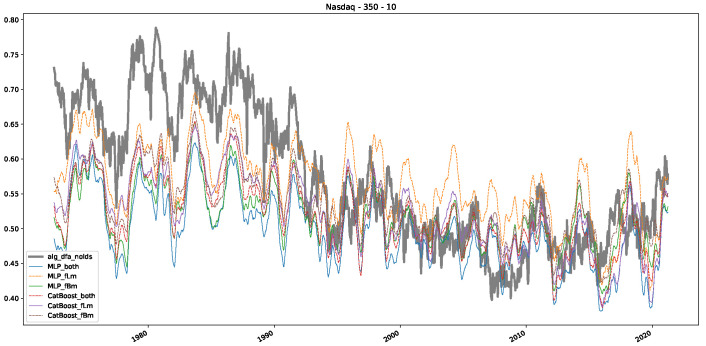
Plot depicting the time-varying DFA and predictions from all trained machine learning models, using a 350-day input window and a 10-day step size between windows for the NASDAQ daily close values.

**Figure A46:**
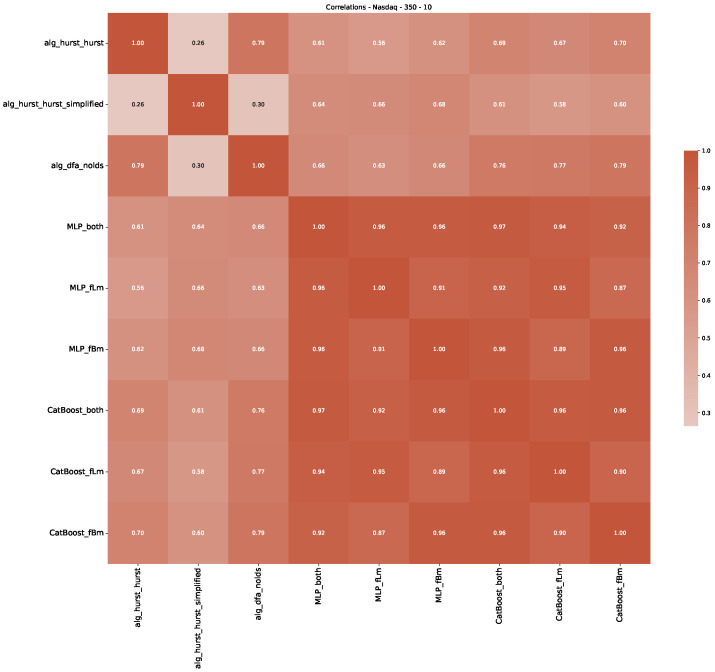
Correlation plot showing the relationships between the DFA, various Hurst exponent estimation methods, and the predictions of all trained machine learning models for the Dow Jones index, using a 350-day rolling window size and a step size of 10 days for the NASDAQ daily close values.

**Figure A47:**
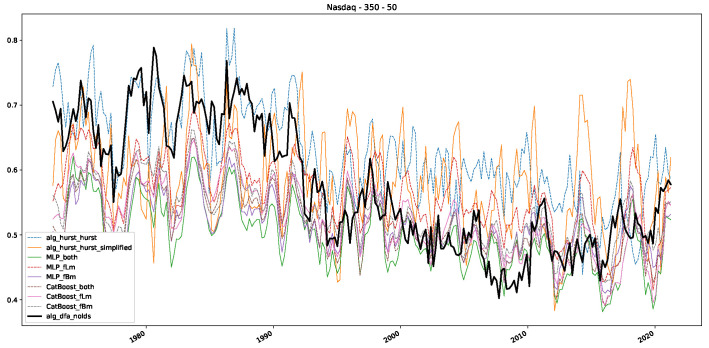
Plot depicting the time-varying DFA and Hurst exponents, as well as the predictions from all trained machine learning models, using a 350-day input window and a 50-day step size between windows for the NASDAQ daily close values.

**Figure A48:**
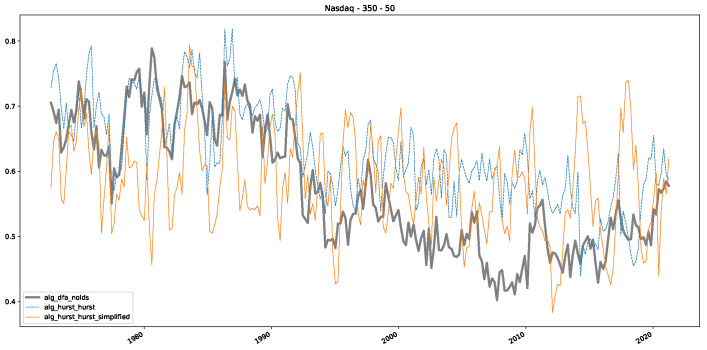
Plot depicting the time-varying DFA and Hurst exponents, using a 350-day input window and a 50-day step size between windows for the NASDAQ daily close values.

**Figure A49:**
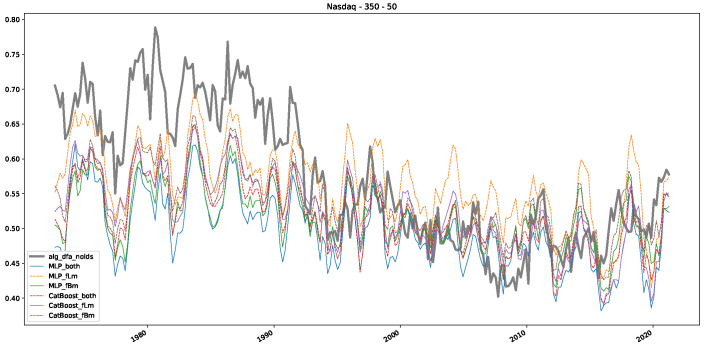
Plot depicting the time-varying DFA and predictions from all trained machine learning models, using a 350-day input window and a 50-day step size between windows for the NASDAQ daily close values.

**Figure A50:**
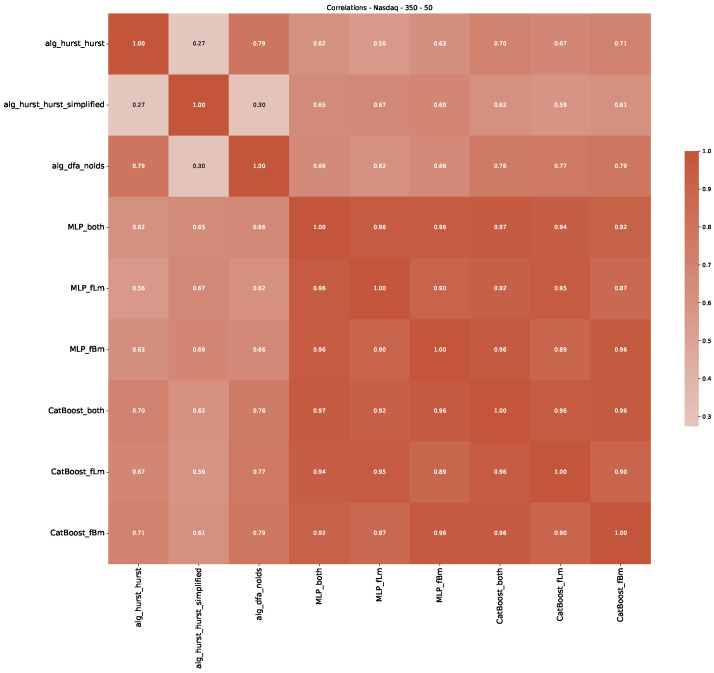
Correlation plot showing the relationships between the DFA, various Hurst exponent estimation methods, and the predictions of all trained machine learning models for the Dow Jones index, using a 350-day rolling window size and a step size of 50 days for the NASDAQ daily close values.

## Data Availability

Our code, the trained models, and our training data are available from our GitHub repository https://github.com/Raubkatz/ML_Hurst_Estimation (accessed on 13 December 2023). The financial data is not available in our repository but can be obtained or bought from various data providers.

## Appendix A. Fractional Lévy Motion and Its Scaling Behavior

This appendix provides several plots that demonstrate how the parameter α in fractional Lévy motions alters the scaling behavior, thereby producing more multifractal signals. We utilized the modified R/S analysis (see Section 5.1) to illustrate this, by plotting the scaling behavior of the fractional Lévy motions under consideration, for three different scaling exponents.

## Appendix B. Additional Plots, Finance Experiments

This appendix provides the additional plots for all discussed assets for the results from Section 5 and Section 5.2.
